# Significance of Th1 and Th2 Cell Densities and Th1/Th2 Cytokine Profiles in Colorectal Cancer

**DOI:** 10.1158/1055-9965.EPI-25-0767

**Published:** 2025-08-14

**Authors:** Aino Ojanperä, Päivi Sirniö, Hanna Elomaa, Ville K. Äijälä, Henna Karjalainen, Vilja V. Tapiainen, Meeri Kastinen, Akseli Kehusmaa, Oskari Rahkola, Vesa-Matti Pohjanen, Maarit Ahtiainen, Olli Helminen, Erkki-Ville Wirta, Jukka Rintala, Sanna Meriläinen, Raila Aro, Reetta Häivälä, Juha Saarnio, Tero Rautio, Toni T. Seppälä, Jan Böhm, Jukka-Pekka Mecklin, Anne Tuomisto, Markus J. Mäkinen, Juha P. Väyrynen

**Affiliations:** 1 Translational Medicine Research Unit, Medical Research Center Oulu, Oulu University Hospital, and University of Oulu, Oulu, Finland.; 2 Research Program in Systems Oncology, University of Helsinki, Helsinki, Finland.; 3 Central Finland Biobank, Hospital Nova of Central Finland, Well Being Services County of Central Finland, Jyväskylä, Finland.; 4 Department of Gastroenterology and Alimentary Tract Surgery, Tampere University Hospital, Tampere, Finland.; 5 Faculty of Medicine and Health Technology, Tampere University and Tays Cancer Centre, Tampere University Hospital, Tampere, Finland.; 6 Department of Gastrointestinal Surgery, Helsinki University Central Hospital, University of Helsinki, Helsinki, Finland.; 7 Applied Tumor Genomics, Research Program Unit, University of Helsinki, Helsinki, Finland.; 8 Department of Pathology, Hospital Nova of Central Finland, Well Being Services County of Central Finland, Jyväskylä, Finland.; 9 Department of Education and Research, Well Being Services County of Central Finland, Jyväskylä, Finland.; 10 Faculty of Sport and Health Sciences, University of Jyväskylä, Jyväskylä, Finland.

## Abstract

**Background:**

T-cell densities are associated with colorectal cancer outcome, but the significance of specific Th cell subsets is incompletely understood. We aimed to investigate the role of Th1 and Th2 cells and associated cytokine profiles.

**Methods:**

We used multiplex IHC to identify Th1 and Th2 cells on tumor samples of more than 2,000 patients with colorectal cancer (three independent cohorts). We measured serum levels of Th1 and Th2 cytokines in mesenteric (*N* = 77) and peripheral (*N* = 603) blood samples. We evaluated the prognostic significance of Th1 and Th2 cells and their associations with circulating cytokines.

**Results:**

High densities of both Th1 and Th2 cells were independently associated with better cancer-specific survival. In the largest cohort, the HR for high vs. low Th1 density was 0.62 (95% confidence interval, 0.44–0.85; *P*_Trend_ = 0.004) and the HR for high vs. low Th2 density was 0.50 [95% confidence interval, 0.36–0.69; *P*_Trend_ < 0.001]. Th1 cell density positively correlated with IL12 levels and negatively with IL10 levels in mesenteric serum. Th1:Th2 cell density ratio positively correlated with mesenteric serum Th1:Th2 produced cytokine ratio.

**Conclusions:**

This study supports the prognostic significance of Th1 and Th2 cells in colorectal cancer. Mesenteric serum levels of Th-related cytokines may contribute to or reflect the tumor-associated Th1/Th2 response.

**Impact:**

Findings highlight comparable roles for Th1 and Th2 cells in predicting favorable outcome and challenge the traditional notion of Th2 cells as tumor promoters.

## Introduction

Colorectal cancer is the third most diagnosed cancer worldwide, and the mortality rate of colorectal cancer is the second highest of all cancers (1). The progression of cancer is influenced not only by genetic or epigenetic alterations driving tumor cell proliferation and invasion but also by environmental factors, such as the local immune response, stromal interactions, growth factors, and other microenvironmental agents (2–4). The tumor microenvironment is a complex network that includes various immune cells, among which T cells play a pivotal role. T cells are broadly categorized into cytotoxic T cells and Th cells, both of which are critical for adaptive immunity. Several studies have highlighted the significance of T cells in colorectal cancer, demonstrating that a strong T-cell infiltrate in tumors is associated with a better clinical outcome in patients with colorectal cancer (5–7). In contrast, weak immune reaction in colorectal cancer is associated with a poor prognosis (8).

Th cells can differentiate into distinct subsets depending on their cytokine environment (9). The six main subtypes, characterized by specific transcription factors and cytokine expression, are Th1, Th2, Th17, regulatory T, Tfh, and Th9 cells (10). Th1 cells are typically associated with antitumor immunity, promoting the activation of cytotoxic T cells by producing cytokines, such as IFN-γ (IFNG), TNF, and IL2 (11). Higher densities of Th1 cells and cytotoxic T cells have been associated with favorable prognosis in colorectal cancer (6, 12, 13). In contrast, Th2 cells have been commonly associated with tumor progression (14). Th2 cells are responsible for humoral immunity and secrete various cytokines, such as IL4, IL5, IL10, and IL13 (11, 15), which can promote carcinogenesis (16, 17). However, some evidence suggests that Th2 cells might also contribute to antitumor immunity through eosinophil recruitment (18, 19) and by remodeling the tumor vasculature (20). Few studies have examined Th2 cell densities in colorectal cancer (13, 21), and the prognostic significance of Th2 cell infiltration as well as Th1:Th2 cell density ratio in colorectal cancer is unclear. Also, the role of cytokines that induce Th1 and Th2 cell polarization or are produced by these cells in the progression of colorectal tumors requires further clarification.

The primary aim of this study was to investigate associations of Th1 and Th2 cell densities with the disease course in colorectal cancer. We hypothesized that higher densities of Th1 and Th2 cells are associated with a stronger immune response and better clinical outcome in colorectal cancer. Our secondary aim was to evaluate the significance of the balance between serum levels of cytokines that these Th cells produce (IFNG, IL2, and TNF for Th1; IL4, IL5, IL10, and IL13 for Th2) and cytokines that guide differentiation of Th1 (IFNG and IL12) and Th2 cells (IL4 and IL33) in colorectal cancer. This included exploring their associations with colorectal cancer prognosis, tumor-infiltrating Th1 and Th2 cell densities, and other tumor and patient characteristics. We hypothesized that elevated levels of Th1-inducing cytokines might indicate an immune response skewed toward Th1 cell production, whereas elevated Th2-inducing cytokines might indicate a shift toward Th2 cell production.

## Materials and Methods

### Study population

Our study population comprised three cohorts (Supplementary Fig. S1A–S1C). All cohorts were analyzed for Th1 and Th2 cell densities, whereas serum samples for cytokine analyses were only available for cohorts 1 and 3.

Survival analyses were conducted for cohorts 1 and 2. Cohort 1 included 1,011 patients with stages I to IV colorectal cancer who underwent surgery between 2006 and 2020 at Oulu University Hospital (22–24). Patients who received chemotherapy or radiotherapy were excluded (*N* = 235), considering the potential effect of neoadjuvant treatment on the immune microenvironment (25). For survival analyses, patients who died less than 30 days after surgery were additionally excluded (*N* = 5 for immune cell analyses and *N* = 4 for serum analyses). Cohort 2 consisted of 1,343 patients with stages I to IV colorectal cancer who underwent surgery between 2000 and 2015 at Central Finland Central Hospital in Jyväskylä (26, 27). As with cohort 1, patients who received chemotherapy or radiotherapy were excluded (*N* = 243), and those who died within 30 days after surgery were excluded from survival analysis (*N* = 37).

Cohort 3 included 77 patients with stages I to IV colon cancer enrolled in the Peri-Nutri Trial who underwent surgery between 2020 and 2024 at Oulu University Hospital (28). Patients in cohort 3 did not receive chemotherapy or radiotherapy, as these treatments are not typically administered for colon adenocarcinoma. Survival analyses were not conducted for this cohort because of the limited follow-up and smaller sample size. Detailed exclusion criteria for cohort 3 are available in the original trial publication (28) and included, among others, recurrent colon adenocarcinoma, disease requiring multiorgan resection, or ineligibility for surgical intervention.

The study was conducted according to the guidelines of the Declaration of Helsinki. For cohort 1, the study was conducted under approval from the Regional Medical Research Ethics Committee of the Wellbeing services county of North Ostrobothnia (25/2002, 42/2005, 122/2009, and 37/2020), Biobank Borealis (BB-2017_1012), and Fimea (FIMEA/2022/001941). All participants gave written informed consent for the study. For cohort 2, the study was conducted under approval from the Regional Medical Research Ethics Committee of the Wellbeing services county of Central Finland (Dnro 13U/2011, 1/2016, 8/2020, and 2/2023), Central Finland Biobank (BB23-0172), and Fimea (Dnro FIMEA/2023/001573, 4/2023). The need to obtain informed consent from the study patients was waived (Dnro FIMEA/2023/001573, 4/2023). For cohort 3, the study was approved by the Ethics Committee at the Oulu University Hospital (235/2018), and all participants signed a written informed consent form.

### Multiplex IHC

Tissue microarrays (TMA) had been constructed from surgical resection specimens. They were designed to contain four cores of 1.0 mm diameter for each tumor, two from the tumor center, and two from the invasive margin. *BRAF* V600E status and mismatch repair (MMR) status were evaluated from the TMA cores by IHC (29, 30).

Two multiplex IHC assays were optimized to detect Th1 and Th2 cells within tumors, with the markers including TBX21 (T-bet), CD3, and CD8 (panel 1 for Th1 cells) and GATA3, CD3, and CD8 (panel 2 for Th2 cells). Although the panels did not include CD4, CD3^+^CD8^−^ cells were considered to mainly correspond to Th cells, given that double-negative (CD3^+^CD4^−^CD8^−^) T cells are scarce, generally representing less than 5% of all T cells (31). The assays were performed with Leica BOND RX IHC stainer (RRID: SCR_025548) using cyclic protocols. After deparaffinization and rehydration, antigen retrieval was performed (BOND Epitope Retrieval Solution 2, Leica AR9640, 30 minutes, 95°C). In the first cycle, the sections were incubated with either TBX21 (Cell Signaling Technology, clone D6N8B, RRID: AB_2616022, panel 1, 1:50) or GATA3 (panel 2, Biocare Medical, clone L50-823, RRID: AB_10895444, 1:50) antibodies that were visualized using BOND Polymer Refine Red Detection Kit (Leica DS9390). In the second cycle, previous antibodies were removed using BOND Epitope Retrieval Solution 1 (Leica AR9961, 30 minutes) at 95°C, and slides were incubated with CD8 antibodies (Leica, 4B11, RRID: AB_3676740, 1:100) that were visualized using BOND Polymer Refine Detection Kit (Leica DS9800). In the third cycle, previous antibodies were removed using BOND Epitope Retrieval Solution 1 (Leica AR9961, 30 minutes) at 95°C, and slides were incubated with CD3 antibodies (Leica, LN10, RRID: AB_563541, 1:100) that were visualized using BOND Polymer Refine Detection Kit (Leica DS9800), with Green Chromogen (Leica DC9913) instead of diaminobenzidine as the chromogen.

### Image analysis

The densities of Th1 and Th2 cells were analyzed with QuPath (RRID: SCR_018257), a software platform for digital pathology and whole-slide image analysis (32). TMA cores were annotated using the *TMA dearrayer* function. A pixel classifier, trained with the *Random trees* algorithm, was used to differentiate tumor epithelial and stromal regions while excluding unrepresentative regions such as whitespace, mucus, necrosis, and folds. Cell detection was performed using the *Cell detection* function, and additional features (Haralick features and smoothed features) were calculated using the *Add intensity features* and *Add smoothed features* commands. An object classifier, also trained with the *Random trees* method, was developed to phenotype cells into the following categories: Th1 cells (panel 1: CD3^+^CD8^−^TBX21^+^), Th2 cells (panel 2: CD3^+^CD8^−^GATA3^+^), other Th cells (panel 1: CD3^+^CD8^−^TBX21^−^, panel 2: CD3^+^CD8^−^GATA3^−^), cytotoxic T cells (CD3^+^CD8^+^), tumor cells (characteristic morphology), and other cells. These functions were combined into an automated script and applied to all images for consistent analysis. Final data are presented as T-cell densities (1/mm^2^). The distributions of Th1 and Th2 cell densities in cohorts 1 and 2 are shown in Supplementary Fig. S2. The analyses were performed blinded to clinical data.

### Serum analysis

Preoperative peripheral venous blood serum samples (abbreviated as peripheral serum throughout the text) were collected from cohort 1 patients, and intraoperative mesenteric venous blood serum samples (abbreviated as mesenteric serum throughout the text) from cohort 3 patients. Serum data were available for 603 patients in cohort 1 and 77 patients in cohort 3. Cytokine levels were measured by Olink Target 96 Immuno-Oncology panel (Olink Proteomics), which utilizes the multiplex proximity extension assay technology and includes the measurements of Th1 and Th2 cytokine levels (IL4, IL5, IL10, IL13, IL33, IL2, TNF, IFNG, and IL12). IL4 values were below limit of detection in 32 samples in cohort 1 and four samples in cohort 3, and these cases were excluded from analyses involving IL4. The protein levels are presented as Olink’s normalized protein expression units on a log_2_ scale.

### Statistical analysis

Statistical analyses were performed using IBM SPSS Statistics for Windows (version 29.0, RRID: SCR_016479). Findings with two-sided *P* < 0.05 were considered statistically significant.

For survival analyses, cell densities and cytokine levels were divided into three equal-sized, ordinal groups (tertiles), based on the 33.3rd and 66.7th percentiles of each marker within each cohort. This categorization produced three risk sets of comparable size, stabilized HR estimates, and enabled graphical presentation of survival differences using Kaplan–Meier curves. Kaplan–Meier estimates and Cox regression models were used for survival analyses. The primary endpoint was cancer-specific survival, defined as the time from surgery to colorectal cancer death. The secondary endpoint was overall survival, defined as the time from surgery to death from any cause. Multivariable Cox proportional hazards regression models were adjusted for the following predetermined covariates consistent with our previous studies (33, 34): sex (male and female), age (<65, 65–75, and >75), year of operation (2000–2005, 2006–2010, 2011–2015, and 2016–2020), tumor location (proximal colon, distal colon, and rectum), disease stage (I–II, III, and IV), tumor grade (low grade and high grade), lymphovascular invasion (negative and positive), MMR status (proficient and deficient), and *BRAF* status (wild-type and mutant). The follow-up was limited to 10-year following surgery, considering that most colorectal cancer deaths occur within this time frame. The median follow-up time for censored cases was 6.0 years (IQR, 3.9–9.7) in cohort 1 and 10.0 years (IQR, 7.3–10.0) in cohort 2, and there were 258 deaths (144 colorectal cancer–associated deaths) in cohort 1 and 520 deaths (290 colorectal cancer–associated deaths) in cohort 2. Two separate cohorts were analyzed in survival analyses to increase the reproducibility and generalizability of the findings.

The associations between cell densities and cytokine levels with clinicopathologic features were analyzed by Mann–Whitney (comparing two classes) or Kruskal–Wallis tests (comparing three or more classes).

The correlations of Th1 and Th2 cell densities in tumor tissue with serum IL2, IL4, IL5, IL10, IL12, IL13, IL33, IFNG, and TNF levels were studied using Pearson’s correlation coefficients and multiple linear regression. In multiple linear regression models, continuous variables were preferred in which it is possible to maintain model precision and reduce information loss (35). Age was treated as a continuous variable, and tumor location and disease stage were dichotomized to reduce model complexity, given the lower sample size in cohort 3. The complete list of covariates was age (continuous), sex (male and female), tumor localization (proximal colon and distal colon/rectum), stage (I–II and III–IV), *BRAF* status (wild-type and mutant), MMR status (proficient and deficient), and tumor grade (low and high). Standardized β coefficients were used to estimate the change in Th1 or Th2 cell density (in SD units) associated with a one-SD increase in the cytokine level, adjusted for all covariates. β values were interpreted as follows: <0.3 = weak, 0.3 to 0.5 = moderate, and >0.5 = strong.

## Results

### Characteristics of Th1 and Th2 infiltrates

**Figure 1.**
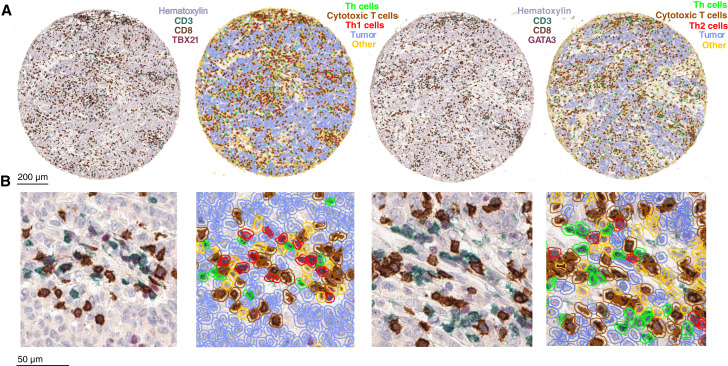
Multiplex IHC assays. The figure shows representative examples of the three-plex IHC assays for the identification of Th1 cells (CD3^+^CD8^−^TBX21^+^), Th2 cells (CD3^+^CD8^−^GATA3^+^), cytotoxic T cells (CD3^+^CD8^+^), and tumor cells. QuPath object classifier result images are represented next to the multiplex IHC images of the same regions. **A,** Low-power images. **B,** High-power images.

Tumor samples of 758 patients with stages I to IV colorectal cancer in cohort 1 and 1,077 (Th1)/1,080 (Th2) patients with stages I to IV colorectal cancer in cohort 2 were successfully analyzed for CD3^+^CD8^−^TBX21^+^ Th1 cell and CD3^+^CD8^−^GATA3^+^ Th2 cell densities using multiplex IHC (Fig. 1A and B). Median Th2 cell densities were 1.7× higher than median Th1 cell densities in cohort 1 and 1.6× higher in cohort 2.

Baseline characteristics of patients according to Th1 and Th2 cell densities are shown in Table 1, and those according to Th1:Th2 cell density ratio in Supplementary Table S1. High Th1 cell densities were associated with female sex, proximal tumor location, low disease stage, high tumor grade, less frequent lymphovascular invasion, MMR-deficient status, and *BRAF* mutation in both cohorts. Similarly, high Th2 cell densities were associated with lower stage and less frequent lymphovascular invasion in both cohorts. Additional associations for high Th2 cell densities included high tumor grade, MMR deficiency, and *BRAF* mutation in cohort 1 and female sex in cohort 2. Tumors with a higher Th1:Th2 cell density ratio were more often MMR-deficient and *BRAF*-mutated, located proximally, high-grade, and categorized in stages II or III in both cohorts.

**Table 1.** Baseline characteristics of patients with colorectal cancer according to Th1 and Th2 cell densities in cohorts 1 and 2.

	Cohort 1					Cohort 2				
Characteristic	Total N	Th1 cell density	*P*	Th2 cell density	*P*	Total *N*^a^	Th1 cell density	*P*	Th2 cell density	*P*
All cases	758 (100%)	68.8 (29.7–135)	​	118 (56.6–214)	​	1,077 (100%)	62.1 (26.9–129)	​	102 (47.3–189)	​
Sex	​	​	0.014	​	0.54	​	​	0.001	​	0.027
Female	356 (47.0%)	80.5 (33.4–145)	​	119 (60.2–212)	​	531 (49.3%)	70.9 (28.1–142)	​	107 (54.8–198)	​
Male	402 (53.0%)	61.3 (26.8–126)	​	115 (53.4–218)	​	546 (50.7%)	52.6 (25.2–113)	​	94.0 (43.6–175)	​
Age (years)	​	​	0.035	​	0.72	​	​	0.061	​	0.65
<65	228 (30.1%)	60.3 (25.2–127)	​	115 (51.6–206)	​	282 (26.2%)	53.8 (24.9–112)	​	97.7 (45.1–184)	​
65–75	279 (36.8%)	68.9 (30.3–131)	​	118 (55.5–214)	​	377 (35.0%)	65.2 (27.7–132)	​	126 (53.7–197)	​
>75	251 (33.1%)	74.3 (33.6–149)	​	121 (57.6–231)	​	418 (38.8%)	66.3 (27.0–136)	​	101 (46.5–187)	​
Tumor location	​	​	<0.001	​	0.061	​	​	0.001	​	0.020
Proximal colon	318 (42.0%)	81.2 (36.6–151)	​	115 (55.1–219)	​	524 (48.7%)	68.9 (30.5–156)	​	106 (50.6–204)	​
Distal colon	203 (26.8%)	50.6 (20.0–109)	​	104 (46.6–198)	​	399 (37.0%)	52.7 (21.2–107)	​	89.6 (42.0–160)	​
Rectum	237 (31.2%)	67.6 (31.9–132)	​	134 (64.4–234)	​	154 (14.3%)	62.3 (30.4–128)	​	114 (58.1–198)	​
AJCC disease stage	​	​	<0.001	​	<0.001	​	​	<0.001	​	<0.001
I	173 (22.8%)	99.9 (48.4–180)	​	171 (85.8–299)	​	179 (16.6%)	87.3 (32.9–186)	​	154 (83.8–306)	​
II	252 (33.3%)	78.9 (35.2–136)	​	119 (61.0–207)	​	399 (37.0%)	76.6 (33.7–147)	​	103 (46.6–188)	​
III	250 (33.0%)	54.0 (27.2–115)	​	112 (60.2–202)	​	349 (32.4%)	50.0 (23.2–104)	​	94.7 (45.9–171)	​
IV	83 (10.9%)	27.0 (7.14–84.1)	​	52.6 (28.1–111)	​	150 (14.0%)	40.9 (14.0–81.1)	​	69.1 (29.7–123)	​
Tumor grade	​	​	0.038	​	0.81	​	​	0.010	​	0.83
Low grade	649 (85.6%)	68.1 (28.0–132)	​	116 (55.5–214)	​	890 (82.6%)	61.3 (26.1–125)	​	102 (48.4–185)	​
High grade	109 (14.4%)	78.6 (39.6–164)	​	138 (58.0–216)	​	187 (17.4%)	70.2 (30.7–166)	​	102 (39.8–208)	​
Lymphovascular invasion	​	​	<0.001	​	<0.001	​	​	<0.001	​	0.014
No	413 (54.5%)	84.9 (37.3–153)	​	136 (63.9–254)	​	836 (77.6%)	64.8 (28.5–136)	​	106 (50.1–192)	​
Yes	345 (45.5%)	52.9 (24.5–110)	​	105 (49.0–182)	​	241 (22.4%)	48.9 (19.9–96.3)	​	86.6 (40.6–168)	​
MMR status	​	​	<0.001	​	<0.001	​	​	<0.001	​	0.75
MMR-proficient	638 (84.2%)	57.3 (26.6–116)	​	111 (53.9–203)	​	916 (85.1%)	56.7 (24.7–115)	​	101 (47.9–188)	​
MMR-deficient	120 (15.8%)	136 (69.5–222)	​	165 (73.2–284)	​	161 (14.9%)	113 (47.4–195)	​	113 (44.6–197)	​
*BRAF* status^b^	​	​	<0.001	​	0.010	​	​	0.001	​	0.73
Wild-type	651 (85.9%)	61.6 (27.5–124)	​	112 (54.4–205)	​	897 (83.4%)	59.6 (25.4–119)	​	103 (48.2–188)	​
Mutant	107 (14.1%)	119 (55.2–203)	​	158 (74.0–269)	​	178 (16.6%)	82.9 (33.1–166)	​	96.3 (42.7–202)	​

NOTE:* P* values were calculated using the Mann–Whitney or Kruskal–Wallis tests.

Abbreviation: AJCC, American Joint Committee on Cancer.

a The patient numbers in the Cohort 2 Total *N* column are for Th1 cell density data. Th2 cell density data were available for 1,080 patients.

b Data are missing from two patients in cohort 2.

### Survival analyses based on Th1 and Th2 cell densities

We first examined the associations of Th cells (CD3^+^CD8^−^) and cytotoxic T cells (CD3^+^CD8^+^) with survival. As expected, higher densities of both were associated with better cancer-specific and overall survival in both cohorts (Supplementary Table S2), with slightly stronger effect size for cytotoxic T cells in cohort 1 but comparable effect size for both in cohort 2.

**Figure 2.**
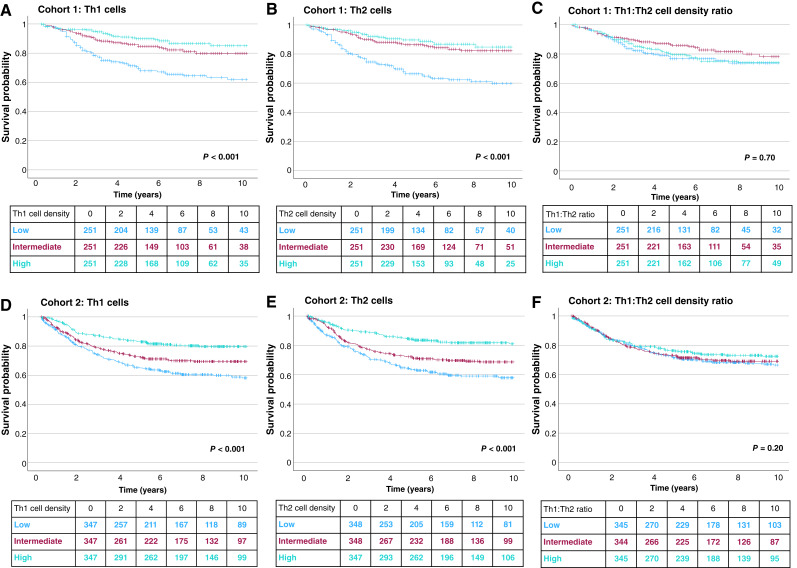
Kaplan–Meier estimates for cancer-specific survival. The Kaplan–Meier curves display cancer-specific survival probability according to Th1 cell densities (**A**), Th2 cell densities, (**B**) and Th1:Th2 cell density ratio (**C**) in cohort 1. **D–F,** Corresponding survival curves for cohort 2. *P* values were calculated using the log-rank test.

Next, we investigated the prognostic value of Th1 cells, Th2 cells, and Th1:Th2 cell density ratio (Fig. 2A–F; Table 2; Supplementary Tables S3 and S4). High Th1 cell density was independently associated with better cancer-specific survival in both cohorts [cohort 1: adjusted HR for high (vs. low) 0.55, 95% confidence interval (Cl), 0.33–0.90, *P*_Trend_ = 0.015; cohort 2: adjusted HR for high (vs. low) 0.62, 95% Cl, 0.44–0.85, *P*_Trend_ = 0.004]. Similar results were found for Th2 cells in both cohorts [cohort 1: adjusted HR for high (vs. low) 0.57, 95% Cl, 0.35–0.92, *P*_Trend_ = 0.004; cohort 2: adjusted HR for high (vs. low) 0.50, 95% Cl, 0.36–0.69, *P*_Trend_ < 0.001]. Conversely, we found no statistically significant associations between Th1:Th2 cell density ratio and cancer-specific survival or overall survival (Table 2).

**Table 2.** Univariable and multivariable Cox regression models for cancer-specific survival and overall survival according to Th1 cell density, Th2 cell density, and Th1:Th2 cell density ratio in cohorts 1 and 2.

	Colorectal cancer–specific survival				Overall survival		
	No. of cases	No. of events	Univariable HR (95% CI)	Multivariable HR (95% CI)	No. of events	Univariable HR (95% CI)	Multivariable HR (95% CI)
Cohort 1	​	​	​	​	​	​	​
Th1 cell density	​	​	​	​	​	​	​
Low	251	78	1 (ref.)	1 (ref.)	113	1 (ref.)	1 (ref.)
Intermediate	251	39	0.46 (0.32–0.68)	0.76 (0.50–1.14)	77	0.63 (0.47–0.84)	0.76 (0.56–1.03)
High	251	27	0.31 (0.20–0.48)	0.55 (0.33–0.90)	68	0.53 (0.39–0.72)	0.65 (0.46–0.90)
*P*_Trend_	​	​	<0.001	0.015	​	<0.001	0.008
Th2 cell density	​	​	​	​	​	​	​
Low	251	82	1 (ref.)	1 (ref.)	123	1 (ref.)	1 (ref.)
Intermediate	251	36	0.38 (0.25–0.56)	0.49 (0.32–0.74)	70	0.47 (0.35–0.64)	0.60 (0.44–0.81)
High	251	26	0.29 (0.19–0.45)	0.57 (0.35–0.92)	65	0.49 (0.36–0.67)	0.72 (0.52–0.99)
*P*_Trend_	​	​	<0.001	0.004	​	<0.001	0.020
Th1:Th2 cell density ratio	​	​	​	​	​	​	​
Low	251	52	1 (ref.)	1 (ref.)	87	1 (ref.)	1 (ref.)
Intermediate	251	39	0.69 (0.45–1.04)	1.18 (0.76–1.85)	75	0.77 (0.56–1.04)	0.90 (0.65–1.24)
High	251	53	0.92 (0.63–1.35)	1.17 (0.77–1.76)	96	0.95 (0.71–1.27)	0.86 (0.63–1.18)
*P*_Trend_	​	​	0.70	0.46	​	0.76	0.35
Cohort 2	​	​	​	​	​	​	​
Th1 cell density	​	​	​	​	​	​	​
Low	347	130	1 (ref.)	1 (ref.)	204	1 (ref.)	1 (ref.)
Intermediate	347	96	0.71 (0.55–0.93)	0.86 (0.66–1.13)	172	0.81 (0.66–0.99)	0.89 (0.73–1.10)
High	347	64	0.43 (0.32–0.58)	0.62 (0.44–0.85)	144	0.61 (0.50–0.76)	0.66 (0.52–0.83)
*P*_Trend_	​	​	<0.001	0.004	​	<0.001	<0.001
Th2 cell density	​	​	​	​	​	​	​
Low	348	130	1 (ref.)	1 (ref.)	205	1 (ref.)	1 (ref.)
Intermediate	348	100	0.70 (0.54–0.91)	0.74 (0.57–0.97)	173	0.76 (0.62–0.93)	0.81 (0.66–0.99)
High	347	58	0.38 (0.28–0.52)	0.50 (0.36–0.69)	141	0.58 (0.47–0.72)	0.64 (0.51–0.79)
*P*_Trend_	​	​	<0.001	<0.001	​	<0.001	<0.001
Th1:Th2 cell density ratio	​	​	​	​	​	​	​
Low	345	103	1 (ref.)	1 (ref.)	180	1 (ref.)	1 (ref.)
Intermediate	344	96	0.94 (0.71–1.25)	1.05 (0.79–1.39)	164	0.93 (0.75–1.15)	1.02 (0.82–1.27)
High	345	86	0.83 (0.62–1.10)	1.11 (0.82–1.50)	171	0.94 (0.76–1.15)	1.05 (0.84–1.31)
*P*_Trend_	​	​	0.20	0.49	​	0.53	0.66

NOTE: Multivariable Cox proportional hazards regression models were adjusted for sex, age (<65, 65–75, and >75), year of operation (2000–2005, 2006–2010, 2011–2015, and 2016–2020), tumor location (proximal colon, distal colon, and rectum), disease stage (I–II, III, and IV), tumor grade (low grade and high grade), lymphovascular invasion (negative and positive), MMR status (proficient and deficient), and *BRAF* status (wild-type and mutant). *P*_trend_ values were calculated by using the three categories of immune cell densities/density ratio as continuous variables in univariable and multivariable Cox proportional hazard regression models.

To gain more detailed understanding on the significance of the spatial distribution of Th1 and Th2 cell infiltrates, their densities were analyzed separately from the tumor epithelial and stromal compartments, as well as from the central area of the tumor and the invasive margin (Supplementary Tables S5 and S6). In cohort 1, Th1 and Th2 cell densities in the tumor center generally showed stronger prognostic value than those measured from the invasive margin, whereas such finding was not as evident in cohort 2. In cohort 1, the strongest effect size was observed for high Th1 cell densities in the tumor epithelial compartment of the tumor center [adjusted HR for high (vs. low) 0.39; 95% Cl, 0.24–0.66; *P*_Trend_ < 0.001; Supplementary Table S5]. In cohort 2, the strongest effect size was observed for Th2 cell densities in the tumor epithelial compartment of the tumor center [adjusted HR for high (vs. low) 0.45, 95% Cl, 0.31–0.64; *P*_Trend_ < 0.001; Supplementary Table S6].

### Tumor-infiltrating Th1 and Th2 cells and systemic Th1–Th2 cytokine environment

To explore the relationship between tumor-infiltrating Th1 and Th2 cell densities and the systemic Th1:Th2 environment, we analyzed serum levels of Th1- and Th2-related cytokines. This included cytokines produced by Th1 cells (IFNG, IL2, and TNF) and Th2 cells (IL4, IL5, IL10, and IL13), along with cytokines that promote differentiation into Th1 cells (IL12 and IFNG) and Th2 cells (IL4 and IL33). In addition to individual cytokine levels, composite indices were calculated to capture the balance of Th1 and Th2 cytokines, including the IFNG:IL4 index, Th1:Th2 produced cytokine index [(IFNG × IL2 × TNF):(IL4 × IL5 × IL10 × IL13)], and Th1:Th2 inducing cytokine index [(IFNG × IL12):(IL4 × IL33)]. For cohort 1, conventional serum samples were obtained from peripheral venous blood. Additionally, in a unique study design enabling the analysis tumor-derived factors before liver processing, mesenteric venous blood serum samples were collected during surgery from 77 patients (cohort 3).

Correlations with mesenteric serum concentrations were first examined (Table 3). Th1 cell densities positively correlated with serum IL12 levels (adjusted β 0.279, *P* = 0.021) and negatively correlated with serum IL10 levels (adjusted β −0.306, *P* = 0.005). No statistically significant correlations were observed between Th2 cell density and mesenteric serum cytokines. The Th1:Th2 cell density ratio positively correlated with the serum Th1:Th2 produced cytokine index (adjusted β 0.306, *P* = 0.014) and serum IL4 levels (adjusted β 0.236, *P* = 0.048).

**Table 3.** Correlations between Th1 and Th2 cell densities in tumors and mesenteric serum cytokine levels in cohort 3.

Mesenteric serum cytokine	*N*	Th1 cell density				Th2 cell density				Th1:Th2 cell density ratio			
		Unadjusted		Adjusted		Unadjusted		Adjusted		Unadjusted		Adjusted	
		Pearson r	*P* value	β	*P* value	Pearson r	*P* value	β	*P* value	Pearson r	*P* value	β	*P* value
IFNG	77^a^	0.203	0.077	0.086	0.48	−0.129	0.27	−0.139	0.27	0.374	<0.001	0.238	0.051
IL12	77^a^	0.351	0.002	0.279	0.021	0.139	0.23	0.144	0.26	0.281	0.014	0.175	0.16
IL2	77^a^	−0.073	0.53	−0.060	0.59	−0.197	0.088	−0.190	0.10	0.103	0.38	0.115	0.31
TNF	77^a^	0.105	0.36	0.032	0.78	−0.157	0.18	−0.181	0.12	0.261	0.023	0.176	0.13
IL33	77^a^	−0.176	0.13	−0.122	0.28	−0.077	0.51	−0.094	0.42	−0.138	0.23	−0.060	0.60
IL4	73^b^	0.023	0.85	0.121	0.30	−0.106	0.38	−0.115	0.35	0.119	0.32	0.236	0.048
IL5	77^a^	−0.009	0.94	−0.28	0.80	0.057	0.62	0.079	0.50	−0.062	0.60	−0.104	0.37
IL10	77^a^	−0.344	0.002	−0.306	0.005	−0.195	0.092	−0.158	0.16	−0.228	0.047	−0.214	0.054
IL13	77^a^	−0.074	0.52	−0.004	0.98	−0.099	0.39	−0.059	0.61	0.008	0.94	0.050	0.66
IFNG:IL4 index	73^b^	0.177	0.13	0.019	0.88	−0.097	0.42	−0.103	0.43	0.320	0.006	0.135	0.30
Th1:Th2-inducing cytokine index	73^b^	0.280	0.016	0.133	0.31	−0.028	0.81	−0.036	0.79	0.370	0.001	0.194	0.15
Th1:Th2 produced cytokine index	73^b^	0.343	0.003	0.233	0.058	−0.008	0.95	−0.029	0.82	0.424	<0.001	0.306	0.014

NOTE: The adjusted β coefficients and *P* values were calculated with linear regression models that included age (continuous), sex (male and female), tumor localization (proximal and distal), stage (I–II and III–IV), *BRAF* status (wild-type and mutant), MMR status (proficient and deficient), and tumor grade (low grade and high grade). Continuous variables that were not normally distributed were logarithmically transformed. The Th1:Th2-inducing cytokine index was based on the formula [(IFNG × IL12):(IL4 × IL33)], and the Th1:Th2 produced cytokine index was based on the formula [(IFNG × IL2 × TNF):(IL4 × IL5 × IL10 × IL13)].

a *N* = 76 for Th2 cell density and Th1:Th2 cell density ratio.

b *N* = 72 for Th2 cell density and Th1:Th2 cell density ratio.

Next, correlations with peripheral serum cytokine levels were analyzed in the larger cohort 1 (Table 4). These correlations were generally weaker than those observed with mesenteric serum cytokines. However, statistically significant, weak correlations were found between Th2 cell density and serum IL12 (adjusted β −0.081, *P* = 0.050), Th2 cell density and the serum Th1:Th2-inducing cytokine index (adjusted β −0.085, *P* = 0.042), Th1:Th2 cell density ratio and serum IFNG (adjusted β 0.126, *P* = 0.002), Th1:Th2 cell density ratio and serum IFNG:IL4 index (adjusted β 0.089, *P* = 0.030), and Th1:Th2 cell density ratio and the serum Th1:Th2-inducing cytokine index (adjusted β 0.086, *P* = 0.039).

**Table 4.** Correlations between Th1 and Th2 cell densities in tumors and serum cytokine levels in cohort 1.

Peripheral serum cytokine	*N*	Th1 cell density				Th2 cell density				Th1:Th2 density ratio			
		Unadjusted		Adjusted		Unadjusted		Adjusted		Unadjusted		Adjusted	
		Pearson r	*P* value	β	*P* value	Pearson r	*P* value	β	*P* value	Pearson r	*P* value	β	*P* value
IFNG	591	0.023	0.57	0.019	0.63	−0.075	0.070	−0.076	0.062	0.131	0.001	0.126	0.002
IL12	591	−0.047	0.25	−0.051	0.20	−0.089	0.031	−0.081	0.050	0.051	0.21	0.035	0.39
IL2	591	−0.073	0.077	−0.062	0.11	0.077	0.060	−0.071	0.080	−0.001	0.98	0.006	0.88
TNF	591	−0.038	0.35	−0.043	0.28	−0.086	0.038	−0.081	0.051	0.058	0.16	0.045	0.27
IL33	591	0.014	0.74	0.026	0.51	−0.017	0.67	−0.016	0.70	0.044	0.28	0.060	0.14
IL4	560	−0.002	0.97	0.021	0.60	−0.020	0.63	−0.005	0.91	0.023	0.59	0.034	0.40
IL5	591	0.039	0.34	0.040	0.31	0.033	0.42	0.032	0.44	0.011	0.78	0.014	0.72
IL10	591	−0.038	0.36	−0.010	0.80	−0.040	0.33	−0.018	0.66	0.0004	0.99	0.010	0.80
IL13	591	−0.026	0.53	−0.025	0.52	−0.044	0.29	−0.047	0.24	0.019	0.64	0.026	0.52
IFNG:IL4 index	560	0.020	0.64	0.008	0.84	−0.053	0.21	−0.059	0.16	0.099	0.019	0.089	0.030
Th1:Th2-inducing cytokine index	560	−0.009	0.83	−0.019	0.63	−0.085	0.045	−0.085	0.042	0.100	0.017	0.086	0.039
Th1:Th2 produced cytokine index	560	−0.020	0.64	−0.029	0.48	−0.056	0.18	−0.059	0.16	0.047	0.26	0.038	0.37

NOTE: The adjusted β coefficients and *P* values were calculated with linear regression models that included age (continuous), sex (male and female), tumor localization (proximal colon and distal colon/rectum), stage (I–II and III–IV), *BRAF* status (wild-type and mutant), MMR status (proficient and deficient), and tumor grade (low grade and high grade). Continuous variables that were not normally distributed were logarithmically transformed. The Th1:Th2-inducing cytokine index was based on the formula [(IFNG × IL12):(IL4 × IL33)], and the Th1:Th2 produced cytokine index was based on the formula [(IFNG × IL2 × TNF):(IL4 × IL5 × IL10 × IL13)].

### Tumor characteristics and serum Th1 and Th2 cytokine levels

The baseline characteristics of tumors according to peripheral serum Th1 cytokine levels and Th2 cytokine levels in cohort 1 are presented in Supplementary Tables S7–S9. Among Th1 cytokines, serum IFNG levels were higher in tumors that were located proximally (*P =* 0.017) and had not invaded into the lymphovascular system (*P =* 0.014). Higher levels of serum IL12 were associated with female sex (*P =* 0.006), older age (*P =* 0.002), and proximal tumor location (*P =* 0.014). Higher serum TNF levels were associated with female sex (*P =* 0.026), older age (*P* < 0.001), *BRAF* mutation (*P =* 0.030), less frequent lymphovascular invasion (*P =* 0.044), and proximal tumor location (*P* < 0.001). Among Th2 cytokines, higher level of serum IL5 was associated with younger age (*P =* 0.049) and tumor location in the distal colon (*P =* 0.024). Serum IL10 levels were higher in older patients (*P =* 0.001) and those with high-stage tumors (*P =* 0.044). Higher levels of serum IL13 were associated with high tumor grade (*P =* 0.031), and higher serum IL33 levels were associated with younger age (*P =* 0.013). Higher serum Th1:Th2 produced cytokine index values were associated with proximal tumor location (*P =* 0.002), whereas higher Th1:Th2 inducing cytokine index values were associated with older age (*P =* 0.020), proximal tumor location (*P =* 0.021), and less frequent lymphovascular invasion (*P =* 0.040).

### Prognostic impact of serum Th1 and Th2 cytokines

To evaluate the potential of circulating Th1 and Th2 related cytokines as colorectal cancer biomarkers, we analyzed their prognostic impact in cohort 1 (Supplementary Table S10). In multivariable models adjusted for common prognostic factors, high serum TNF levels were associated with worse overall survival [adjusted HR for high (vs. low) 1.49; 95% Cl, 1.01–2.20; *P*_Trend_ = 0.045], high serum IL5 levels were associated with better cancer-specific survival [adjusted HR for high (vs. low) 0.61; 95% Cl, 0.38–0.99; *P*_Trend_ = 0.045], and a higher Th1:Th2 produced cytokine index was associated with worse cancer-specific survival [adjusted HR for high (vs. low) 1.71; 95% Cl, 1.04–2.81; *P*_Trend_ = 0.036]. However, these associations were considerably weaker than those observed for Th1 and Th2 cell densities in the tumor microenvironment.

## Discussion

This study builds on the established understanding on the critical role of T cells in colorectal cancer prognosis, providing new insights into the specific contributions of Th1 and Th2 subtypes. Notably, high densities of both Th1 cells and Th2 cells were strongly associated with better cancer-specific survival and overall survival, underscoring their shared prognostic value. Whereas systemic levels of cytokines produced by these subsets seem to play a less direct role in survival, our data indicate that Th1- and Th2-related cytokines, as detected from mesenteric serum, may contribute to or reflect the tumor-associated Th1/Th2 response. This study stands out because of the unprecedented size and scope of its cohorts, making a significant advancement in the evaluation of Th cell dynamics in colorectal cancer.

Precise identification of Th1 and Th2 cells is an essential component of this study. Whereas TBX21 and GATA3 are generally considered relatively specific biomarkers to Th1 and Th2 cells, they are also expressed in other cell types, such as NK cells (36), cytotoxic T cells (36, 37), B cells, and innate lymphoid cells (38). To minimize potential misidentification, we utilized robust multiplex IHC protocols incorporating CD3, a pan-T cell marker, and CD8, a marker specific to cytotoxic T cells. Th1 and Th2 cells were defined as those coexpressing TBX21 or GATA3 with CD3 but lacking CD8 expression. CD8 was included in the panel instead of CD4, considering that double-negative T cells are rare (31), CD4 is also expressed by macrophages (39), and inclusion of CD4 would have resulted in overlapping chromogenic signals (brown along with red and green) in Th1 and Th2 cells, complicating their accurate identification.

Higher densities of both Th1 and Th2 cells were more often found in MMR-deficient tumors compared with MMR-proficient tumors, supporting the notion that a high mutational burden in these tumors elicits a strong immune response, which is also accompanied by better prognosis (14, 40). The favorable prognostic role of Th1 cells aligns with findings from earlier studies published over the past two decades (6, 7, 13). Interestingly, the strongest associations were found in the tumor center, whereas slightly weaker associations were noted at the invasive margin. This suggests that the Th1 infiltration may play a more significant role in the tumor center, a trend also observed for Th2 cells. Compared with several previous studies, our study benefits from the multimarker approach for cell phenotyping which offered enhanced specificity. For example, TBX21 alone is insufficient as a marker for Th1 cells, as it is also expressed by populations of cytotoxic T cells and NK cells (41, 42).

The role of Th2 cells in the progression of colorectal cancer has remained controversial, with some studies suggesting tumor-promoting effects and others proposing a protective role (43, 44). For example, Tosolini and colleagues (13) reported that Th2 cluster gene expression was not predictive of clinical outcome in colorectal cancer. In contrast, our study showed that high densities of Th2 cells were associated with better prognosis of colorectal cancer. Considering the role of Th2 cells and cytokines in B-cell proliferation and eosinophil recruitment (45), it is possible that the favorable role of Th2 cells is, in part, mediated through interactions with these cell types, both of which have been associated with favorable outcome in colorectal cancer (46–48). Additionally, Th2 cells may contribute to protective tumor vasculature remodeling, as discussed before (20). Our findings indicate that the prognostic value of Th2 cells is comparable with that of Th1 cells, and both subsets show potential to be incorporated into clinical tools for predicting colorectal cancer outcome in the future. Notably, analyses of the Th1:Th2 cell density ratio revealed no evidence that a Th1-dominant response is more advantageous for tumor suppression compared with a Th2-dominant response. These results challenge the notion that Th2 cells are primarily tumor-promoting (14) and suggest that this concept warrants reevaluation.

The cytokines analyzed in this study were categorized into those produced by Th1 and Th2 cells and those involved in guiding their differentiation. The analyses were implemented on both mesenteric serum and peripheral serum, aiming to identify markers that remain detectable after hepatic metabolism and have prognostic clinical significance. In peripheral blood analyses, we found only a few weak correlations with local Th cell densities. However, in mesenteric blood, which is closer to the tumor site and potentially reflects tumor-derived cytokines more directly, more significant correlations were detected. For instance, Th1 cell densities positively correlated with mesenteric serum IL12 levels, and Th1:Th2 cell density ratio correlated with the mesenteric serum Th1:Th2 produced cytokine index. These findings suggest that cytokines secreted in the tumor microenvironment may influence mesenteric serum cytokine levels, reflecting Th1 cell infiltration or the Th1:Th2 balance within the tumor. Despite these correlations, the associations between peripheral serum cytokine levels and survival were weak, highlighting the importance of direct tumor microenvironment assessments over systemic evaluations for prognostic purposes.

From a biomarker perspective, further studies should investigate whether quantitative assessment of intratumoral Th1 and Th2 cells by multiplex IHC could complement the CD3/CD8-based Immunoscore, which is already clinically validated for prognostication in stages II to III colorectal cancer and is under prospective evaluation in clinical trials (49, 50). Therapeutically, our observation that both Th1 and Th2 infiltrates are associated with favorable outcome suggests that complete depletion of Th2 responses may be counterproductive. Rather, these results support immunomodulatory approaches that preserve or restore a balanced Th cell response. Whereas Th1-promoting strategies, such as tumor-targeted IL12 (51), remain a promising area of development, therapeutic approaches that enhance Th2-mediated antitumor mechanisms, including eosinophil and B-cell activation, also warrant further exploration.

There are some limitations in this study. The cell density analysis was based on TMAs, which may not fully capture the heterogeneity of the tumor microenvironment. However, we mitigated this limitation by analyzing several TMA cores from each patient, sampling both the tumor center and the invasive margin. We excluded patients who received neoadjuvant treatment from the study, which primarily included rectal tumors. Hence, rectal tumors were underrepresented, and the prognostic significance of Th1 and Th2 cells in preoperatively treated tumors remain unclear. Another limitation lies in the genetic homogeneity of the study population, as the patients were predominantly White and treated in Finnish hospitals. The generalizability of the findings to other populations requires further investigation. Moreover, this study was based on multiplex IHC analysis of Th1 and Th2 cell densities and serum-based assessment of related cytokines. Incorporating tumor RNA sequencing or spatial transcriptomic data could provide complementary evidence regarding immune cell activation states and cytokine expression. Such approaches may offer deeper mechanistic insights in future studies. Lastly, this study focused exclusively on Th1 and Th2 cells, leaving the roles of other immune cells unexamined. The strengths of this study include two large colorectal cancer cohorts and a unique smaller cohort with mesenteric serum samples. Extensive clinicopathologic data allowed us to adjust survival models for multiple variables such as tumor location, tumor grade, disease stage, lymphovascular invasion, MMR status, and *BRAF* status, strengthening the relevance of the findings.

In conclusion, this study supports the prognostic significance of both Th1 and Th2 cells in colorectal cancer, highlighting their comparable roles in predicting favorable outcome and challenging the traditional notion of Th2 cells as tumor promoters. Although local Th cell densities showed some correlations with mesenteric serum Th1/Th2 cytokine levels, systemic Th1/Th2 cytokine measurements showed limited prognostic utility. Further research into the interplay between these immune components and validation in diverse populations is needed to translate these insights into clinical practice.

## Supplementary Material

Figure S1 Flow charts of the three patient cohorts analyzed in the study. The figure illustrates the number of patients included in the various analyses for Cohort 1 (A), Cohort 2 (B), and Cohort 3 (C).

Figure S2 Distribution of Th1 and Th 2 cell densities. Histograms illustrate the distribution of Th1 and Th2 cell densities (cells/mm2) in Cohorts 1 and 2.

Table S1 Baseline characteristics of colorectal cancer patients according to Th1:Th2 cell density ratio in Cohorts 1 and 2.

Table S2 Univariable and multivariable Cox regression models for cancer-specific survival and overall survival according to CD3+CD8- T cell and CD3+CD8+ T cell densities in Cohorts 1 and 2.

Table S3 Multivariable Cox regression models for Th1 and Th2 cell densities and patient survival in Cohort 1.

Table S4 Multivariable Cox regression models for Th1 and Th2 cell densities and patient survival in Cohort 2.

Table S5 Univariable and multivariable Cox regression models for cancer-specific survival and overall survival according to Th1 cell density and Th2 cell density in the tumor epithelial and stromal compartment of the tumor center and the invasive margin in Cohort 1.

Table S6 Univariable and multivariable Cox regression models for cancer-specific survival and overall survival according to Th1 cell density and Th2 cell density in the tumor epithelial and stromal compartment of the tumor center and the invasive margin in Cohort 2.

Table S7 Baseline characteristics of colorectal cancer patients according to serum Th1 cytokine levels in Cohort 1.

Table S8 Baseline characteristics of colorectal cancer patients according to serum Th2 cytokine levels in Cohort 1.

Table S9 Baseline characteristics of colorectal cancer patients according to serum Th1:Th2 cytokine indices in Cohort 1.

Table S10 Univariable and multivariable Cox regression models for cancer-specific survival and overall survival according to serum cytokine levels in Cohort 1.

## Data Availability

Data generated and/or analyzed during this study are not publicly available. The sharing of data will require approval from relevant ethics committees and/or biobanks. Further information including the procedures to obtain and access data of Finnish Biobanks is described at https://finbb.fi/en/fingenious-service.
