# Supplementary material for: Significance of Th1 and Th2 Cell Densities and Th1/Th2 Cytokine Profiles in Colorectal Cancer
Source: Cancer Epidemiol Biomarkers Prev. 2025 Aug 14;34(11):2032–41. doi: 10.1158/1055-9965.EPI-25-0767 (PMC12580825; doi:10.1158/1055-9965.EPI-25-0767)
Supplement: Figure S1 — Flow charts of the three patient cohorts analyzed in the study. The figure illustrates the number of patients included in the various analyses for Cohort 1 (A), Cohort 2 (B), and Cohort 3 (C). [file epi-25-0767_figure_s1_suppsf1.pdf]

A

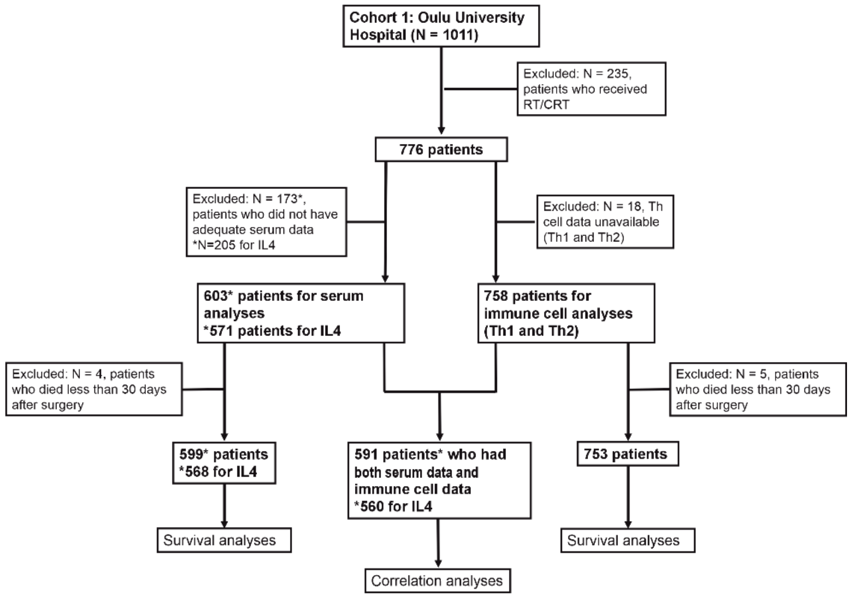

B

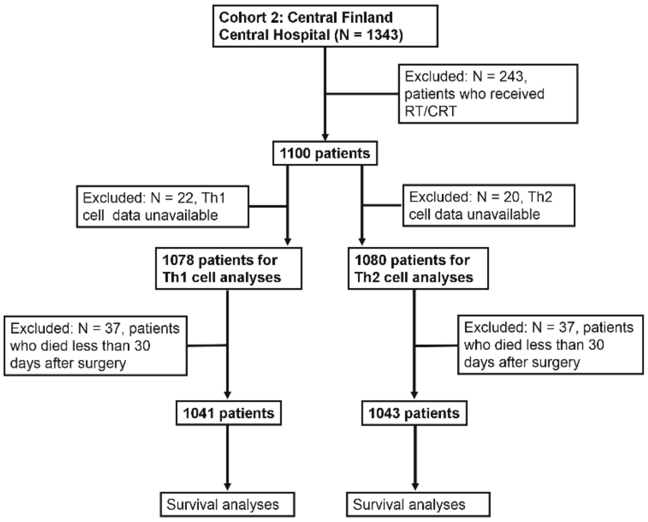

C

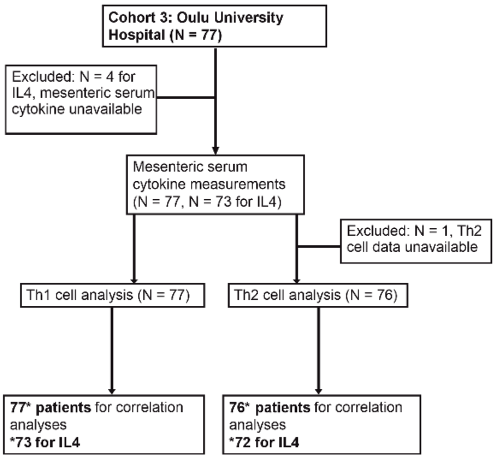

Figure S1. Flow charts of the three patient cohorts analyzed in the study. The figure illustrates the number of patients included in the various analyses for Cohort 1 (A), Cohort 2 (B), and Cohort 3 (C).
