# Supplementary material for: Significance of Th1 and Th2 Cell Densities and Th1/Th2 Cytokine Profiles in Colorectal Cancer
Source: Cancer Epidemiol Biomarkers Prev. 2025 Aug 14;34(11):2032–41. doi: 10.1158/1055-9965.EPI-25-0767 (PMC12580825; doi:10.1158/1055-9965.EPI-25-0767)
Supplement: Figure S2 — Distribution of Th1 and Th 2 cell densities. Histograms illustrate the distribution of Th1 and Th2 cell densities (cells/mm2) in Cohorts 1 and 2. [file epi-25-0767_figure_s2_suppsf2.pdf]

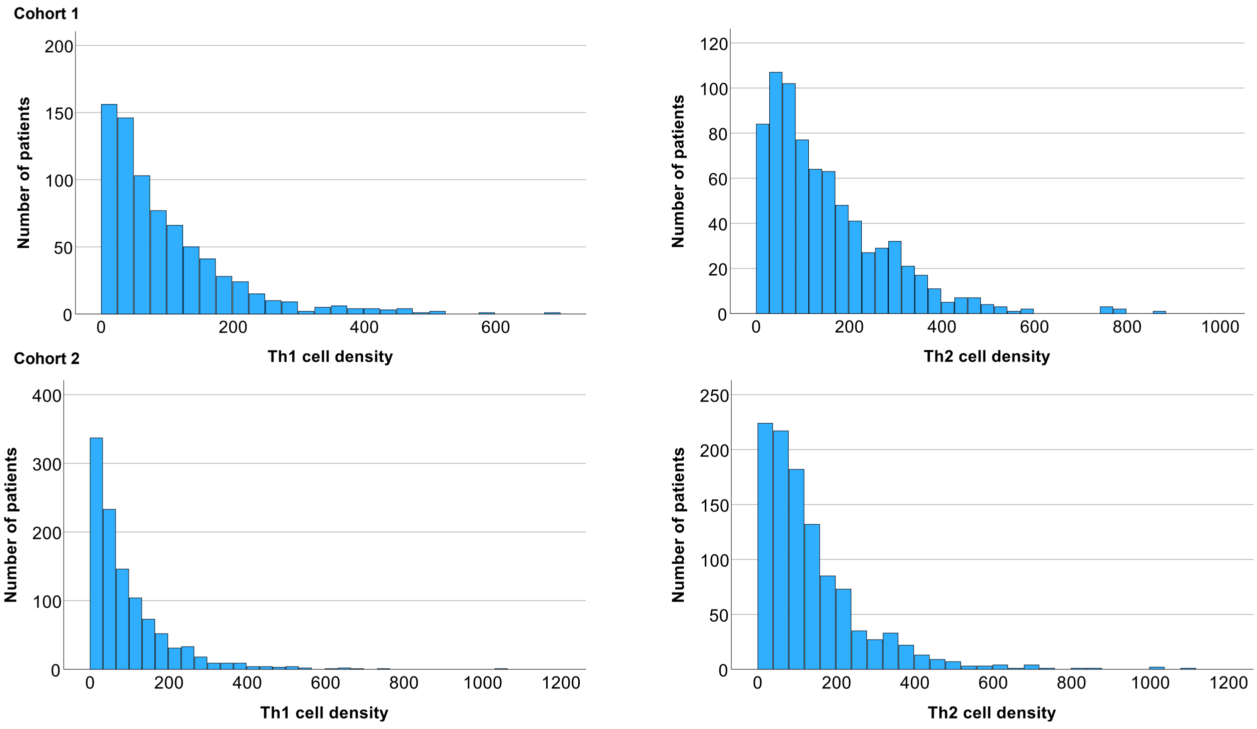

Figure S2. Distribution of Th1 and Th 2 cell densities. Histograms illustrate the distribution of Th1 and Th2 cell densities (cells/mm^2) in Cohorts 1 and 2.
