# Supplementary material for: Significance of Th1 and Th2 Cell Densities and Th1/Th2 Cytokine Profiles in Colorectal Cancer
Source: Cancer Epidemiol Biomarkers Prev. 2025 Aug 14;34(11):2032–41. doi: 10.1158/1055-9965.EPI-25-0767 (PMC12580825; doi:10.1158/1055-9965.EPI-25-0767)
Supplement: Table S1 — Baseline characteristics of colorectal cancer patients according to Th1:Th2 cell density ratio in Cohorts 1 and 2. [file epi-25-0767_table_s1_suppst1.pdf]

Table S1. Baseline characteristics of colorectal cancer patients according to Th1:Th2 cell density ratio in Cohorts 1 and 2.

Characteristic	Cohort 1 Total N	Cohort 1 Th1:Th2 cell density ratio	Cohort 1 *P*	Cohort 2 Total N	Cohort 2 Th1:Th2 cell density ratio	Cohort 2 *P*
All cases	758 (100.0%)	0.590 (0.366–0.918)		1070 (100.0 %)	0.646 (0.363–1.08)	
Sex			0.003			0.124
Female	356 (47.0 %)	0.642 (0.397–0.977)		526 (49.2 %)	0.676 (0.357–1.10)	
Male	402 (53.0 %)	0.551 (0.335–0.865)		544 (50.8 %)	0.620 (0.365–1.01)	
Age (years)			0.007			0.056
<65	228 (30.1 %)	0.547 (0.312–0.889)		279 (26.1 %)	0.607 (0.319–0.985)	
65–75	279 (36.8 %)	0.578 (0.374–0.889)		375 (35.0 %)	0.639 (0.370–1.08)	
>75	251 (33.1 %)	0.648 (0.417–0.972)		416 (38.9 %)	0.676 (0.383–1.14)	
Tumor location			< 0.001			0.002
Proximal colon	318 (42.0 %)	0.687 (0.421–1.04)		521 (48.7 %)	0.727 (0.380–1.16)	
Distal colon	203 (26.8 %)	0.550 (0.332–0.790)		396 (37.0 %)	0.613 (0.345–0.999)	
Rectum	237 (31.2 %)	0.529 (0.341–0.853)		153 (14.3 %)	0.531 (0.354–0.872)	
AJCC disease stage			0.004			< 0.001
I	173 (22.8 %)	0.588 (0.364–0.893)		179 (16.7 %)	0.583 (0.319–0.947)	
II	252 (33.3 %)	0.645 (0.411–1.05)		399 (37.3 %)	0.722 (0.440–1.20)	
III	250 (33.0 %)	0.563 (0.351–0.830)		347 (32.4 %)	0.626 (0.350–0.999)	
IV	83 (10.9 %)	0.455 (0.274–0.917)		145 (13.6 %)	0.592 (0.315–0.972)	
Tumor grade			0.004			< 0.001
Low-grade	649 (85.6 %)	0.565 (0.363-0.889)		885 (82.7 %)	0.613 (0.345-1.03)	
High-grade	109 (14.4 %)	0.749 (0.406–1.11)		185 (17.3 %)	0.780 (0.475–1.28)	
Lymphovascular invasion			0.051			0.093
No	413 (54.5 %)	0.609 (0.377–0.936)		830 (77.6 %)	0.652 (0.371–1.11)	
Yes	345 (45.5 %)	0.563 (0.347–0.883)		240 (22.4 %)	0.623 (0.334–0.968)	
MMR status			< 0.001			< 0.001
MMR proficient	638 (84.2 %)	0.536 (0.342–0.835)		909 (85.0 %)	0.603 (0.342–0.978)	
MMR deficient	120 (15.8 %)	0.903 (0.657–1.36)		161 (15.0 %)	1.02 (0.649–1.67)	
BRAF status[a]			< 0.001			< 0.001
Wild-type	651 (85.9 %)	0.558 (0.348–0.889)		891 (83.4 %)	0.620 (0.349–0.998)	
Mutant	107 (14.1 %)	0.756 (0.561–1.20)		177 (16.6 %)	0.928 (0.480–1.31)	

[a]Data missing from two patients in Cohort 2. Abbreviations: AJCC, American Joint Committee on Cancer; MMR, mismatch repair. *P* values were calculated using the Mann-Whitney or Kruskal-Wallis test.
