# Supplementary material for: Significance of Th1 and Th2 Cell Densities and Th1/Th2 Cytokine Profiles in Colorectal Cancer
Source: Cancer Epidemiol Biomarkers Prev. 2025 Aug 14;34(11):2032–41. doi: 10.1158/1055-9965.EPI-25-0767 (PMC12580825; doi:10.1158/1055-9965.EPI-25-0767)
Supplement: Table S2 — Univariable and multivariable Cox regression models for cancer-specific survival and overall survival according to CD3+CD8- T cell and CD3+CD8+ T cell densities in Cohorts 1 and 2. [file epi-25-0767_table_s2_suppst2.pdf]

Table S2. Univariable and multivariable Cox regression models for cancer-specific survival and overall survival according to CD3$^+$CD8$^-$ T cell and CD3$^+$CD8$^+$ T cell densities in Cohorts 1 and 2.

	No. of cases	Colorectal cancer-specific survival			Overall survival		
		No. of events	Univariable HR (95% CI)	Multivariable HR (95% CI)	No. of events	Univariable HR (95% CI)	Multivariable HR (95% CI)
**Cohort 1**							
**CD3$^+$CD8$^-$ T cells**							
Low	251	75	1 (referent)	1 (referent)	112	1 (referent)	1 (referent)
Intermediate	251	43	0.51 (0.35-0.74)	0.90 (0.60-1.34)	84	0.65 (0.49-0.87)	0.91 (0.67-1.23)
High	251	26	0.31 (0.20-0.48)	0.54 (0.33-0.88)	62	0.48 (0.35-0.66)	0.67 (0.48-0.93)
P_{Trend}			< 0.001	0.016		< 0.001	0.018
**CD3$^+$CD8$^+$ T cells**							
Low	251	85	1 (referent)	1 (referent)	113	1 (referent)	1 (referent)
Intermediate	251	39	0.43 (0.29-0.63)	0.69 (0.46-1.04)	87	0.71 (0.53-0.93)	0.81 (0.60-1.09)
High	251	20	0.22 (0.13-0.35)	0.37 (0.22-0.64)	58	0.48 (0.35-0.66)	0.55 (0.39-0.78)
P_{Trend}			< 0.001	< 0.001		< 0.001	< 0.001
**Cohort 2**							
**CD3$^+$CD8$^-$ T cells**							
Low	350	137	1 (referent)	1 (referent)	211	1 (referent)	1 (referent)
Intermediate	350	97	0.66 (0.51-0.85)	0.92 (0.70-1.20)	176	0.77 (0.63-0.94)	0.87 (0.71-1.07)
High	350	59	0.37 (0.27-0.50)	0.51 (0.37-0.70)	137	0.55 (0.44-0.68)	0.60 (0.48-0.75)
P_{Trend}			< 0.001	< 0.001		< 0.001	< 0.001
**CD3$^+$CD8$^+$ T cells**							
Low	350	139	1 (referent)	1 (referent)	201	1 (referent)	1 (referent)
Intermediate	350	98	0.67 (0.52-0.87)	0.71 (0.54-0.92)	181	0.86 (0.70-1.05)	0.83 (0.67-1.02)
High	350	56	0.35 (0.26-0.48)	0.50 (0.36-0.69)	142	0.61 (0.50-0.76)	0.65 (0.62-0.82)
P_{Trend}			< 0.001	< 0.001		< 0.001	< 0.001

Abbreviations: CI, confidence interval; HR, hazard ratio
Multivariable Cox proportional hazards regression models were adjusted for sex, age (<65, 65–75, >75), year of operation (2000–2005, 2006–2010, 2011–2015, 2016–2020), tumor location (proximal colon, distal colon, rectum), disease stage (I–II, III, IV), tumor grade (low-grade, high-grade), lymphovascular invasion (negative, positive), mismatch repair (MMR) status (proficient, deficient), *BRAF* status (wild-type, mutant).
