# Supplementary material for: Significance of Th1 and Th2 Cell Densities and Th1/Th2 Cytokine Profiles in Colorectal Cancer
Source: Cancer Epidemiol Biomarkers Prev. 2025 Aug 14;34(11):2032–41. doi: 10.1158/1055-9965.EPI-25-0767 (PMC12580825; doi:10.1158/1055-9965.EPI-25-0767)
Supplement: Table S3 — Multivariable Cox regression models for Th1 and Th2 cell densities and patient survival in Cohort 1. [file epi-25-0767_table_s3_suppst3.pdf]

Table S3. Multivariable Cox regression models for Th1 and Th2 cell densities and patient survival in Cohort 1.

	Th1 cell density		Th2 cell density	
	Cancer-specific survival	Overall survival	Cancer-specific survival	Overall survival
	Multivariable HR (95% CI)	Multivariable HR (95% CI)	Multivariable HR (95% CI)	Multivariable HR (95% CI)
Cell density				
Low	1 (referent)	1 (referent)	1 (referent)	1 (referent)
Intermediate	0.76 (0.50-1.14)	0.76 (0.56-1.03)	0.49 (0.32-0.74)	0.60 (0.44-0.81)
High	0.55 (0.33-0.90)	0.65 (0.46-0.90)	0.57 (0.35-0.92)	0.72 (0.52-0.99)
Age				
<65	1 (referent)	1 (referent)	1 (referent)	1 (referent)
65–75	1.60 (1.04-2.47)	1.67 (1.16-2.40)	1.58 (1.03-2.42)	1.61 (1.12-2.32)
>75	2.46 (1.58-3.82)	4.23 (3.00-5.97)	2.33 (1.51-3.61)	3.99 (2.84-5.62)
Sex				
Male	1 (referent)	1 (referent)	1 (referent)	1 (referent)
Female	1.00 (0.71-1.40)	0.82 (0.63-1.05)	1.08 (0.77-1.52)	0.85 (0.66-1.10)
Year of operation				
2006–2010	1 (referent)	1 (referent)	1 (referent)	1 (referent)
2010–2015	0.86 (0.57-1.30)	0.83 (0.62-1.13)	0.88 (0.59-1.31)	0.87 (0.64-1.17)
2016–2020	0.54 (0.34-0.83)	0.62 (0.45-0.86)	0.57 (0.36-0.88)	0.66 (0.47-0.91)
Tumor location				
Proximal colon	1 (referent)	1 (referent)	1 (referent)	1 (referent)
Distal colon	1.16 (0.75-1.79)	1.09 (0.78-1.53)	1.19 (0.77-1.82)	1.09 (0.78-1.52)
Rectum	0.94 (0.60-1.45)	1.05 (0.75-1.45)	0.97 (0.63-1.50)	1.05 (0.76-1.46)
AJCC stage				
I–II	1 (referent)	1 (referent)	1 (referent)	1 (referent)
III	2.60 (1.55-4.37)	1.23 (0.89-1.70)	2.68 (1.59-4.50)	1.27 (0.92-1.75)
IV	16.94 (9.68-29.63)	7.41 (5.04-10.90)	16.01 (9.12-28.12)	7.34 (4.98-10.81)
Tumor grade				
Low-grade	1 (referent)	1 (referent)	1 (referent)	1 (referent)
High-grade	1.49 (0.97-2.28)	1.16 (0.82-1.65)	1.41 (0.92-2.17)	1.12 (0.78-1.59)
Lymphovascular invasion				
No	1 (referent)	1 (referent)	1 (referent)	1 (referent)
Yes	2.07 (1.30-3.30)	1.42 (1.05-1.92)	2.20 (1.38-3.50)	1.48 (1.10-2.00)
MMR status				
MMR proficient	1 (referent)	1 (referent)	1 (referent)	1 (referent)
MMR deficient	0.57 (0.27-1.21)	1.09 (0.69-1.72)	0.51 (0.24-1.09)	0.99 (0.62-1.56)
BRAF mutation				
Wild type	1 (referent)	1 (referent)	1 (referent)	1 (referent)
Mutant	1.95 (1.06-3.58)	1.48 (0.96-2.28)	1.87 (1.03-3.41)	1.43 (0.92-2.20)

Abbreviations: AJCC, American Joint Committee on Cancer; CI, confidence interval; HR, hazard ratio; MMR, mismatch repair.
