# Supplementary material for: Significance of Th1 and Th2 Cell Densities and Th1/Th2 Cytokine Profiles in Colorectal Cancer
Source: Cancer Epidemiol Biomarkers Prev. 2025 Aug 14;34(11):2032–41. doi: 10.1158/1055-9965.EPI-25-0767 (PMC12580825; doi:10.1158/1055-9965.EPI-25-0767)
Supplement: Table S4 — Multivariable Cox regression models for Th1 and Th2 cell densities and patient survival in Cohort 2. [file epi-25-0767_table_s4_suppst4.pdf]

Table S4. Multivariable Cox regression models for Th1 and Th2 cell densities and patient survival in Cohort 2.

	Th1 cell density		Th2 cell density	
	Cancer-specific survival	Overall survival	Cancer-specific survival	Overall survival
	Multivariable HR (95% CI)	Multivariable HR (95% CI)	Multivariable HR (95% CI)	Multivariable HR (95% CI)
Cell density				
Low	1 (referent)	1 (referent)	1 (referent)	1 (referent)
Intermediate	0.86 (0.66-1.13)	0.89 (0.73-1.10)	0.74 (0.57-0.97)	0.81 (0.66-0.99)
High	0.62 (0.44-0.85)	0.66 (0.52-0.83)	0.50 (0.36-0.69)	0.64 (0.51-0.79)
Age				
<65	1 (referent)	1 (referent)	1 (referent)	1 (referent)
65–75	1.16 (0.86-1.57)	1.31 (1.02-1.68)	1.15 (0.85-1.55)	1.31 (1.02-1.68)
>75	1.89 (1.41-2.54)	3.04 (2.40-3.83)	1.92 (1.42-2.58)	3.05 (2.41-3.85)
Sex				
Male	1 (referent)	1 (referent)	1 (referent)	1 (referent)
Female	0.90 (0.70-1.15)	0.77 (0.64-0.93)	0.89 (0.70-1.14)	0.75 (0.63-0.90)
Year of operation				
2000–2005	1 (referent)	1 (referent)	1 (referent)	1 (referent)
2006–2010	0.57 (0.43-0.76)	0.63 (0.51-0.79)	0.57 (0.43-0.76)	0.65 (0.53-0.80)
2011–2015	0.47 (0.35-0.63)	0.59 (0.48-0.74)	0.47 (0.35-0.63)	0.59 (0.47-0.73)
Tumor location				
Proximal colon	1 (referent)	1 (referent)	1 (referent)	1 (referent)
Distal colon	0.86 (0.66-1.12)	0.93 (0.76-1.14)	0.83 (0.64-1.09)	0.91 (0.74-1.11)
Rectum	0.81 (0.56-1.18)	0.87 (0.65-1.15)	0.84 (0.58-1.22)	0.88 (0.67-1.17)
AJCC stage				
I–II	1 (referent)	1 (referent)	1 (referent)	1 (referent)
III	2.90 (2.10-4.00)	1.42 (1.15-1.75)	2.96 (2.15-4.06)	1.45 (1.18-1.79)
IV	17.16 (12.18-24.17)	7.69 (6.00-9.87)	16.51 (11.69-23.32)	7.59 (5.90-9.76)
Tumor grade				
Low-grade	1 (referent)	1 (referent)	1 (referent)	1 (referent)
High-grade	1.95 (1.44-2.65)	1.97 (1.56-2.49)	1.92 (1.42-2.61)	1.97 (1.56-2.49)
Lymphovascular invasion				
No	1 (referent)	1 (referent)	1 (referent)	1 (referent)
Yes	1.85 (1.44-2.39)	1.58 (1.29-1.94)	1.96 (1.52-2.52)	1.63 (1.33-2.00)
MMR status				
MMR proficient	1 (referent)	1 (referent)	1 (referent)	1 (referent)
MMR deficient	0.52 (0.31-0.88)	0.70 (0.49-1.00)	0.48 (0.29-0.81)	0.64 (0.45-0.90)
BRAF mutation				
Wild type	1 (referent)	1 (referent)	1 (referent)	1 (referent)
Mutant	1.42 (0.93-2.18)	1.47 (1.07-2.03)	1.40 (0.92-2.15)	1.48 (1.08-2.02)

Abbreviations: AJCC, American Joint Committee on Cancer; CI, confidence interval; HR, hazard ratio; MMR, mismatch repair.
