# Supplementary material for: Significance of Th1 and Th2 Cell Densities and Th1/Th2 Cytokine Profiles in Colorectal Cancer
Source: Cancer Epidemiol Biomarkers Prev. 2025 Aug 14;34(11):2032–41. doi: 10.1158/1055-9965.EPI-25-0767 (PMC12580825; doi:10.1158/1055-9965.EPI-25-0767)
Supplement: Table S5 — Univariable and multivariable Cox regression models for cancer-specific survival and overall survival according to Th1 cell density and Th2 cell density in the tumor epithelial and stromal compartment of the tumor center and the invasive margin in Cohort 1. [file epi-25-0767_table_s5_suppst5.pdf]

Table S5. Univariable and multivariable Cox regression models for cancer-specific survival and overall survival according to Th1 cell density and Th2 cell density in the tumor epithelial and stromal compartment of the tumor center and the invasive margin in Cohort 1.

		Colorectal cancer-specific survival			Overall survival		
	No. of cases	No. of events	Univariable HR (95% CI)	Multivariable HR (95% CI)	No. of events	Univariable HR (95% CI)	Multivariable HR (95% CI)
**Th1 cell density, tumor, central**							
Low	250	81	1 (referent)	1 (referent)	113	1 (referent)	1 (referent)
Intermediate	250	34	0.36 (0.24-0.54)	0.44 (0.29-0.67)	70	0.52 (0.39-0.70)	0.55 (0.41-0.76)
High	250	28	0.29 (0.19-0.45)	0.39 (0.24-0.66)	73	0.53 (0.39-0.71)	0.49 (0.35-0.69)
P_{Trend}			< 0.001	< 0.001		< 0.001	< 0.001
**Th1 cell density, tumor, invasive margin**							
Low	241	67	1 (referent)	1 (referent)	99	1 (referent)	1 (referent)
Intermediate	242	35	0.47 (0.31-0.70)	0.80 (0.52-1.22)	63	0.55 (0.40-0.75)	0.73 (0.52-1.01)
High	242	36	0.49 (0.33-0.74)	0.92 (0.57-1.47)	87	0.78 (0.59-1.04)	0.83 (0.60-1.16)
P_{Trend}			< 0.001	0.60		0.094	0.26
**Th1 cell density, stroma, central**							
Low	250	80	1 (referent)	1 (referent)	114	1 (referent)	1 (referent)
Intermediate	250	33	0.37 (0.24-0.55)	0.52 (0.34-0.79)	75	0.59 (0.44-0.79)	0.63 (0.47-0.85)
High	250	30	0.34 (0.22-0.51)	0.51 (0.32-0.82)	67	0.54 (0.40-0.72)	0.63 (0.46-0.87)
P_{Trend}			< 0.001	0.001		< 0.001	0.002
**Th1 cell density, stroma, invasive margin**							
Low	242	72	1 (referent)	1 (referent)	105	1 (referent)	1 (referent)
Intermediate	242	38	0.51 (0.34-0.75)	1.04 (0.67-1.62)	78	0.71 (0.53-0.95)	0.91 (0.66-1.25)
High	241	28	0.36 (0.23-0.56)	0.77 (0.46-1.28)	66	0.59 (0.43-0.80)	0.77 (0.55-1.10)
P_{Trend}			< 0.001	0.37		< 0.001	0.15
**Th2 cell density, tumor, central**							
Low	452	102	1 (referent)	1 (referent)	164	1 (referent)	1 (referent)
Intermediate	149	23	0.66 (0.42-1.03)	0.95 (0.60-1.53)	44	0.79 (0.56-1.10)	0.96 (0.68-1.35)
High	148	18	0.51 (0.31-0.83)	0.52 (0.30-0.91)	48	0.83 (0.60-1.14)	0.83 (0.59-1.17)
P_{Trend}			0.003	0.034		0.16	0.31
**Th2 cell density, tumor, invasive margin**							
Low	597	122	1 (referent)	1 (referent)	206	1 (referent)	1 (referent)
Intermediate	65	9	0.67 (0.34-1.31)	0.71 (0.35-1.44)	18	0.80 (0.49-1.29)	0.92 (0.56-1.51)
High	64	7	0.52 (0.24-1.11)	0.92 (0.42-2.02)	23	0.98 (0.64-1.51)	1.14 (0.73-1.80)
P_{Trend}			0.048	0.52		0.68	0.69
**Th2 cell density, stroma, central**							
Low	250	83	1 (referent)	1 (referent)	121	1 (referent)	1 (referent)
Intermediate	249	38	0.42 (0.29-0.62)	0.50 (0.34-0.76)	81	0.62 (0.47-0.82)	0.76 (0.57-1.02)
High	250	22	0.25 (0.16-0.40)	0.45 (0.27-0.73)	54	0.43 (0.31-0.60)	0.64 (0.46-0.90)
P_{Trend}			< 0.001	< 0.001		< 0.001	0.007
**Th2 cell density, stroma, invasive margin**							
Low	242	70	1 (referent)	1 (referent)	102	1 (referent)	1 (referent)
Intermediate	242	45	0.62 (0.43-0.90)	0.80 (0.54-1.19)	84	0.79 (0.59-1.06)	0.88 (0.65-1.19)
High	242	23	0.31 (0.20-0.50)	0.66 (0.39-1.11)	61	0.59 (0.43-0.82)	0.83 (0.59-1.18)
P_{Trend}			< 0.001	0.10		0.001	0.28

Abbreviations: CI, confidence interval; HR, hazard ratio

Multivariable Cox proportional hazards regression models were adjusted for sex, age (<65, 65–75, >75), year of operation (2000–2005, 2006–2010, 2011–2015, 2016–2020), tumor location (proximal colon, distal colon, rectum), disease stage (I–II, III, IV), tumor grade (low-grade, high-grade), lymphovascular invasion (negative, positive), mismatch repair (MMR) status (proficient, deficient), *BRAF* status (wild-type, mutant).
