# Supplementary material for: Significance of Th1 and Th2 Cell Densities and Th1/Th2 Cytokine Profiles in Colorectal Cancer
Source: Cancer Epidemiol Biomarkers Prev. 2025 Aug 14;34(11):2032–41. doi: 10.1158/1055-9965.EPI-25-0767 (PMC12580825; doi:10.1158/1055-9965.EPI-25-0767)
Supplement: Table S6 — Univariable and multivariable Cox regression models for cancer-specific survival and overall survival according to Th1 cell density and Th2 cell density in the tumor epithelial and stromal compartment of the tumor center and the invasive margin in Cohort 2. [file epi-25-0767_table_s6_suppst6.pdf]

Table S6. Univariable and multivariable Cox regression models for cancer-specific survival and overall survival according to Th1 cell density and Th2 cell density in the tumor epithelial and stromal compartment of the tumor center and the invasive margin in Cohort 2.

		Colorectal cancer-specific survival			Overall survival		
	No. of cases	No. of events	Univariable HR (95% CI)	Multivariable HR (95% CI)	No. of events	Univariable HR (95% CI)	Multivariable HR (95% CI)
**Th1 cell density, tumor, central**							
Low	341	126	1 (referent)	1 (referent)	208	1 (referent)	1 (referent)
Intermediate	342	84	0.59 (0.45-0.78)	0.83 (0.62-1.10)	156	0.65 (0.53-0.80)	0.79 (0.63-0.97)
High	341	73	0.52 (0.39-0.69)	0.67 (0.49-0.92)	148	0.63 (0.51-0.78)	0.66 (0.52-0.83)
P_{Trend}			< 0.001	0.013		< 0.001	< 0.001
**Th1 cell density, tumor, invasive margin**							
Low	315	124	1 (referent)	1 (referent)	182	1 (referent)	1 (referent)
Intermediate	315	68	0.51 (0.38-0.69)	0.69 (0.50-0.94)	141	0.73 (0.58-0.90)	0.85 (0.68-1.07)
High	315	73	0.56 (0.42-0.75)	0.87 (0.62-1.22)	148	0.78 (0.63-0.97)	0.86 (0.67-1.11)
P_{Trend}			< 0.001	0.25		0.023	0.22
**Th1 cell density, stroma, central**							
Low	341	137	1 (referent)	1 (referent)	208	1 (referent)	1 (referent)
Intermediate	342	84	0.55 (0.42-0.72)	0.75 (0.56-0.99)	161	0.68 (0.55-0.84)	0.76 (0.62-0.94)
High	341	62	0.38 (0.28-0.51)	0.53 (0.39-0.73)	143	0.56 (0.45-0.69)	0.64 (0.51-0.80)
P_{Trend}			< 0.001	< 0.001		< 0.001	< 0.001
**Th1 cell density, stroma, invasive margin**							
Low	315	126	1 (referent)	1 (referent)	189	1 (referent)	1 (referent)
Intermediate	315	80	0.58 (0.44-0.77)	0.85 (0.64-1.14)	149	0.72 (0.58-0.89)	0.88 (0.70-1.09)
High	315	59	0.41 (0.30-0.56)	0.64 (0.45-0.89)	133	0.62 (0.50-0.77)	0.72 (0.56-0.91)
P_{Trend}			< 0.001	0.009		< 0.001	0.007
**Th2 cell density, tumor, central**							
Low	485	167	1 (referent)	1 (referent)	268	1 (referent)	1 (referent)
Intermediate	272	74	0.75 (0.57-0.99)	0.86 (0.65-1.14)	137	0.85 (0.69-1.04)	0.93 (0.75-1.15)
High	271	40	0.36 (0.25-0.51)	0.45 (0.31-0.64)	107	0.56 (0.45-0.71)	0.64 (0.50-0.80)
P_{Trend}			< 0.001	< 0.001		< 0.001	< 0.001
**Th2 cell density, tumor, invasive margin**							
Low	656	213	1 (referent)	1 (referent)	356	1 (referent)	1 (referent)
Intermediate	150	26	0.45 (0.30-0.68)	0.71 (0.46-1.08)	58	0.57 (0.43-0.76)	0.76 (0.57-1.02)
High	149	23	0.41 (0.26-0.62)	0.53 (0.34-0.83)	62	0.63 (0.48-0.82)	0.64 (0.48-0.85)
P_{Trend}			< 0.001	0.002		< 0.001	< 0.001
**Th2 cell density, stroma, central**							
Low	343	127	1 (referent)	1 (referent)	203	1 (referent)	1 (referent)
Intermediate	343	94	0.69 (0.53-0.90)	0.78 (0.59-1.02)	164	0.74 (0.60-0.91)	0.83 (0.67-1.02)
High	342	60	0.40 (0.30-0.55)	0.54 (0.39-0.74)	145	0.60 (0.49-0.75)	0.70 (0.56-0.87)
P_{Trend}				< 0.001		< 0.001	0.001
**Th2 cell density, stroma, invasive margin**							
Low	318	124	1 (referent)	1 (referent)	192	1 (referent)	1 (referent)
Intermediate	319	89	0.66 (0.50-0.87)	0.85 (0.64-1.13)	163	0.76 (0.62-0.94)	0.90 (0.72-1.11)
High	318	49	0.33 (0.24-0.46)	0.54 (0.38-0.76)	121	0.52 (0.41-0.65)	0.62 (0.49-0.79)
P_{Trend}			< 0.001	< 0.001		< 0.001	< 0.001

Abbreviations: CI, confidence interval; HR, hazard ratio

Multivariable Cox proportional hazards regression models were adjusted for sex, age (<65, 65–75, >75), year of operation (2000–2005, 2006–2010, 2011–2015, 2016–2020), tumor location (proximal colon, distal colon, rectum), disease stage (I–II, III, IV), tumor grade (low-grade, high-grade), lymphovascular invasion (negative, positive), mismatch repair (MMR) status (proficient, deficient), *BRAF* status (wild-type, mutant).
