# Supplementary material for: Significance of Th1 and Th2 Cell Densities and Th1/Th2 Cytokine Profiles in Colorectal Cancer
Source: Cancer Epidemiol Biomarkers Prev. 2025 Aug 14;34(11):2032–41. doi: 10.1158/1055-9965.EPI-25-0767 (PMC12580825; doi:10.1158/1055-9965.EPI-25-0767)
Supplement: Table S7 — Baseline characteristics of colorectal cancer patients according to serum Th1 cytokine levels in Cohort 1. [file epi-25-0767_table_s7_suppst7.pdf]

Table S7. Baseline characteristics of colorectal cancer patients according to serum Th1 cytokine levels in Cohort 1.

Characteristic	Total N	Serum IFNG	*P*	Serum IL12	*P*	Serum IL2	*P*	Serum TNF	*P*
All cases	603 (100 %)	7.82 (7.17–8.69)		7.31 (6.83–7.82)		0.546 (0.326–0.788)		4.54 (4.25–4.85)	
Sex			0.63		0.006		0.36		0.026
Female	274 (45.4 %)	7.86 (7.16–8.71)		7.39 (6.90–7.89)		0.537 (0.347–0.795)		4.58 (4.29–4.90)	
Male	329 (54.6 %)	7.80 (7.17–8.68)		7.23 (6.72–7.75)		0.553 (0.302–0.774)		4.52 (4.20–4.81)	
Age (years)			0.37		0.002		0.42		< 0.001
<65	174 (28.8 %)	7.74 (6.96–8.54)		7.13 (6.66–7.67)		0.523 (0.324–0.773)		4.33 (4.13–4.65)	
65–75	226 (37.5 %)	7.91 (7.17–8.68)		7.33 (6.85–7.86)		0.566 (0.344–0.794)		4.56 (4.28–4.87)	
>75	203 (33.7 %)	7.82 (7.21–8.85)		7.44 (6.86–8.00)		0.536 (0.276–0.785)		4.69 (4.40–5.00)	
Tumor location			0.017		0.014		0.86		< 0.001
Proximal colon	250 (41.4 %)	8.01 (7.30–8.83)		7.40 (6.89–7.93)		0.556 (0.308–0.786)		4.60 (4.31–4.94)	
Distal colon	165 (27.4 %)	7.84 (7.18–8.85)		7.31 (6.85–7.82)		0.551 (0.350–0.793)		4.61 (4.26–4.86)	
Rectum	188 (31.2 %)	7.61 (7.05–8.50)		7.20 (6.70–7.66)		0.536 (0.310–0.793)		4.45 (4.16–4.69)	
AJCC disease stage			0.70		0.32		0.16		0.15
I	155 (25.7 %)	7.67 (7.17–8.77)		7.26 (6.65–7.76)		0.490 (0.310–0.785)		4.47 (4.19–4.84)	
II	186 (30.9 %)	7.96 (7.16–8.79)		7.32 (6.74–7.83)		0.553 (0.336–0.788)		4.54 (4.26–4.85)	
III	204 (33.8 %)	7.82 (7.25–8.55)		7.32 (6.90–7.85)		0.562 (0.288–0.788)		4.55 (4.26–4.82)	
IV	58 (9.6 %)	7.94 (6.89–8.50)		7.36 (6.98–7.92)		0.642 (0.449–0.902)		4.65 (4.32–5.00)	
Tumor grade			0.59		0.61		0.50		0.98
Low-grade	515 (85.4 %)	7.84 (7.16-8.69)		7.31 (6.83-7.82)		0.543 (0.319-0.789)		4.55 (4.24-4.85)	
High-grade	88 (14.6 %)	7.68 (7.17–8.84)		7.39 (6.81–7.93)		0.572 (0.379–0.786)		4.52 (4.25–4.83)	
Lymphovascular invasion			0.014		0.29		0.49		0.044
No	335 (55.6 %)	7.95 (7.29–8.81)		7.34 (6.86–7.83)		0.535 (0.318–0.788)		4.56 (4.29–4.89)	
Yes	268 (44.4 %)	7.76 (6.98–8.45)		7.30 (6.78–7.81)		0.584 (0.331–0.790)		4.54 (4.19–4.82)	
MMR status			0.76		0.59		0.40		0.65
MMR proficient	506 (83.9 %)	7.83 (7.17–8.71)		7.31 (6.84–7.80)		0.550 (0.334–0.793)		4.54 (4.25–4.85)	
MMR deficient	97 (16.1 %)	7.67 (7.16–8.61)		7.38 (6.67–7.95)		0.536 (0.273–0.761)		4.58 (4.26–4.92)	
BRAF status[a]			0.60		0.37		0.42		0.030
Wild-type	510 (85.4 %)	7.82 (7.16–8.71)		7.31 (6.84–7.82)		0.537 (0.326–0.788)		4.54 (4.23–4.85)	
Mutant	87 (14.6 %)	7.83 (7.35–8.72)		7.36 (6.74–7.88)		0.586 (0.339–0.802)		4.63 (4.34–4.98)	

[a]Data missing from six patients (597 patients in total). Abbreviations: AJCC, American Joint Committee on Cancer; MMR, mismatch repair. *P* values were calculated using the Mann-Whitney or Kruskal-Wallis test.
