# Supplementary material for: Significance of Th1 and Th2 Cell Densities and Th1/Th2 Cytokine Profiles in Colorectal Cancer
Source: Cancer Epidemiol Biomarkers Prev. 2025 Aug 14;34(11):2032–41. doi: 10.1158/1055-9965.EPI-25-0767 (PMC12580825; doi:10.1158/1055-9965.EPI-25-0767)
Supplement: Table S8 — Baseline characteristics of colorectal cancer patients according to serum Th2 cytokine levels in Cohort 1. [file epi-25-0767_table_s8_suppst8.pdf]

Table S8. Baseline characteristics of colorectal cancer patients according to serum Th2 cytokine levels in Cohort 1.

Characteristic	Total N	Serum IL4[b]	*P*	Serum IL5	*P*	Serum IL10	*P*	Serum IL13	*P*	Serum IL33	*P*
All cases	603 (100 %)	1.98 (1.50–2.63)		0.792 (0.456–1.37)		3.60 (3.28–3.97)		1.03 (0.667–1.45)		-0.886 (-1.03– -0.726)	
Sex			1.00		0.94		0.14		0.77		0.049
Female	274 (45.4 %)	2.00 (1.49–2.60)		0.799 (0.447–1.34)		3.61 (3.34–4.03)		1.02 (0.687–1.44)		-0.902 (-1.05– -0.767)	
Male	329 (54.6 %)	1.97 (1.50–2.65)		0.791 (0.464–1.40)		3.58 (3.22–3.94)		1.03 (0.645–1.50)		-0.877 (-1.01– -0.706)	
Age (years)			0.56		0.049		0.001		0.28		0.013
<65	174 (28.8 %)	1.97 (1.56–2.52)		0.926 (0.500–1.66)		3.51 (3.16–3.85)		1.05 (0.747–1.51)		-0.855 (-0.970– -0.691)	
65–75	226 (37.5 %)	1.94 (1.51–2.53)		0.788 (0.435–1.20)		3.61 (3.26–3.99)		0.987 (0.593–1.38)		-0.886 (-1.06– -0.752)	
>75	203 (33.7 %)	2.08 (1.49–2.75)		0.719 (0.432–1.33)		3.66 (3.39–4.06)		1.06 (0.667–1.52)		-0.924 (-1.04– -0.737)	
Tumor location			0.72		0.024		0.58		0.38		0.062
Proximal colon	250 (41.4 %)	1.98 (1.49–2.60)		0.714 (0.431–1.21)		3.63 (3.30–3.97)		1.01 (0.610–1.42)		-0.918 (-1.04– -0.772)	
Distal colon	165 (27.4 %)	2.00 (1.55–2.63)		0.919 (0.526–1.59)		3.57 (3.30–4.00)		1.02 (0.690–1.43)		-0.841 (-1.00– -0.672)	
Rectum	188 (31.2 %)	1.95 (1.44–2.65)		0.795 (0.450–1.52)		3.58 (3.23–3.96)		1.07 (0.738–1.53)		-0.884 (-1.04– -0.737)	
AJCC disease stage			0.33		0.77		0.044		0.58		0.075
I	155 (25.7 %)	2.00 (1.51–2.68)		0.792 (0.443–1.23)		3.54 (3.21–3.86)		1.01 (0.610–1.40)		-0.894 (-1.07– -0.755)	
II	186 (30.9 %)	1.92 (1.49–2.51)		0.791 (0.459–1.53)		3.60 (3.31–3.96)		1.01 (0.637–1.51)		-0.874 (-1.01– -0.723)	
III	204 (33.8 %)	2.00 (1.49–2.65)		0.781 (0.468–1.34)		3.60 (3.31–4.00)		1.05 (0.691–1.46)		-0.901 (-1.06– -0.731)	
IV	58 (9.6 %)	2.12 (1.62–2.66)		0.909 (0.454–1.70)		3.72 (3.37–4.50)		1.03 (0.782–1.41)		-0.850 (-0.954– -0.699)	
Tumor grade			0.35		0.66		0.66		0.031		0.37
Low-grade	515 (85.4 %)	2.00 (1.50-2.65)		0.791 (0.455-1.37)		3.60 (3.26-3.98)		1.01 (0.644-1.41)		-0.886 (-1.04- -0.724)	
High-grade	88 (14.6 %)	1.91 (1.49–2.42)		0.805 (0.498–1.41)		3.62 (3.35–3.95)		1.24 (0.749–1.59)		-0.870 (-0.974– -0.734)	
Lymphovascular invasion			0.27		0.76		0.53		0.68		0.48
No	335 (55.6 %)	1.99 (1.54–2.68)		0.798 (0.461–1.39)		3.61 (3.27–3.98)		1.03 (0.667–1.51)		-0.886 (-1.03– -0.728)	
Yes	268 (44.4 %)	1.98 (1.44–2.48)		0.787 (0.429–1.34)		3.58 (3.29–3.97)		1.03 (0.670–1.41)		-0.884 (-1.02– -0.719)	
MMR status			0.52		0.50		0.60		0.86		0.32
MMR proficient	506 (83.9 %)	2.00 (1.50–2.62)		0.797 (0.468–1.37)		3.60 (3.27–3.98)		1.02 (0.670–1.45)		-0.882 (-1.02– -0.724)	
MMR deficient	97 (16.1 %)	1.93 (1.50–2.67)		0.773 (0.407–1.41)		3.60 (3.30–3.91)		1.09 (0.624–1.46)		-0.935 (-1.04– -0.751)	
BRAF status[a]			0.52		0.69		0.22		0.73		0.96
Wild-type	510 (85.4 %)	1.98 (1.50–2.64)		0.797 (0.459–1.37)		3.59 (3.27–3.96)		1.03 (0.672–1.47)		-0.885 (-1.03– -0.728)	
Mutant	87 (14.6 %)	1.95 (1.50–2.56)		0.773 (0.422–1.38)		3.72 (3.29–4.00)		1.11 (0.560–1.45)		-0.886 (-1.01– -0.684)	

[a]Data missing from six patients (597 patients in total). [b]Data missing from 32 patients (571 patients in total). Abbreviations: AJCC, American Joint Committee on Cancer; MMR, mismatch repair. *P* values were calculated using the Mann-Whitney or Kruskal-Wallis test.
