# Supplementary material for: Significance of Th1 and Th2 Cell Densities and Th1/Th2 Cytokine Profiles in Colorectal Cancer
Source: Cancer Epidemiol Biomarkers Prev. 2025 Aug 14;34(11):2032–41. doi: 10.1158/1055-9965.EPI-25-0767 (PMC12580825; doi:10.1158/1055-9965.EPI-25-0767)
Supplement: Table S9 — Baseline characteristics of colorectal cancer patients according to serum Th1:Th2 cytokine indices in Cohort 1. [file epi-25-0767_table_s9_suppst9.pdf]

Table S9. Baseline characteristics of colorectal cancer patients according to serum Th1:Th2 cytokine indices in Cohort 1.

Characteristic	Total N	IFNG:IL4 index	*P*	Th1:Th2 produced cytokine index [(IFNG×IL2×TNF):(IL4×IL5×IL10×IL13)]	*P*	Th1:Th2 inducing cytokine index [(IFNG×IL12):(IL4×IL33)]	*P*
All cases	571 (100 %)	5.51 (4.73–6.38)		4.24 (2.98–5.58)		8.81 (8.11–9.66)	
Sex			0.79		0.86		0.060
Female	256 (44.8 %)	5.57 (4.71–6.45)		4.15 (2.96–5.58)		8.97 (8.19–9.77)	
Male	315 (55.2 %)	5.47 (4.73–6.38)		4.26 (2.99–5.55)		8.74 (8.07–9.48)	
Age (years)			0.49		0.055		0.020
<65	166 (29.1 %)	5.41 (4.68–6.22)		3.93 (2.74–5.38)		8.57 (7.94–9.41)	
65–75	215 (37.6 %)	5.49 (4.88–6.38)		4.42 (3.06–5.74)		8.85 (8.13–9.73)	
>75	190 (33.3 %)	5.63 (4.60–6.51)		4.32 (3.08–5.54)		8.95 (8.24–9.84)	
Year of operation			0.78		0.72		0.79
2000–2005	-	-		-		-	
2006–2010	26 (4.6 %)	5.44 (4.37–6.35)		4.53 (3.11–5.89)		8.69 (8.01–10.03)	
2011–2015	193 (33.8 %)	5.46 (4.51–6.48)		4.12 (2.97–5.42)		8.77 (8.05–9.70)	
2016–2020	352 (61.6 %)	5.55 (4.88–6.36)		4.32 (2.98–5.58)		8.85 (8.16–9.59)	
Tumor location			0.065		0.002		0.021
Proximal colon	235 (41.2 %)	5.76 (4.84–6.58)		4.61 (3.26–5.78)		9.03 (8.16–9.90)	
Distal colon	156 (27.3 %)	5.52 (4.73–6.45)		4.00 (2.77–5.40)		8.73 (8.10–9.73)	
Rectum	180 (31.5 %)	5.29 (4.65–6.14)		4.03 (2.71–5.07)		8.57 (8.05–9.34)	
AJCC disease stage			0.53		0.45		0.49
I	147 (25.7 %)	5.51 (4.49–6.35)		4.26 (2.99–5.73)		8.68 (8.03–9.47)	
II	175 (30.6 %)	5.65 (4.86–6.55)		4.49 (2.99–5.68)		9.00 (8.07–9.93)	
III	191 (33.5 %)	5.41 (4.78–6.34)		4.17 (2.95–5.38)		8.78 (8.18–9.48)	
IV	58 (10.2 %)	5.31 (4.70–6.20)		3.93 (2.69–5.60)		8.76 (8.10–9.74)	
Tumor grade			0.84		0.34		0.77
Low-grade	487 (85.3 %)	5.53 (4.72-6.40)		4.24 (3.05-5.58)		8.80 (8.12-9.67)	
High-grade	84 (14.7 %)	5.48 (4.74–6.34)		4.09 (2.67–5.62)		8.86 (8.03–9.61)	
Lymphovascular invasion			0.059		0.058		0.040
No	317 (55.5 %)	5.67 (4.86–6.48)		4.49 (3.03–5.70)		8.92 (8.14–9.76)	
Yes	254 (44.5 %)	5.35 (4.66–6.30)		4.09 (2.88–5.39)		8.63 (8.09–9.47)	
MMR status			0.80		0.34		0.73
MMR proficient	478 (83.7 %)	5.50 (4.73–6.36)		4.14 (2.98–5.55)		8.80 (8.12–9.59)	
MMR deficient	93 (16.3 %)	5.51 (4.71–6.56)		4.60 (2.97–5.82)		8.85 (8.04–9.90)	
BRAF status[a]			0.26		0.24		0.25
Wild-type	484 (84.8 %)	5.48 (4.73–6.37)		4.15 (2.95–5.55)		8.79 (8.11–9.56)	
Mutant	82 (14.4 %)	5.71 (4.82–6.53)		4.61 (3.24–5.73)		9.05 (8.15–9.94)	

[a]Data missing from five patients (566 patients in total). Abbreviations: AJCC, American Joint Committee on Cancer; MMR, mismatch repair. *P* values were calculated using the Mann-Whitney or Kruskal-Wallis test.
