# Supplementary material for: Significance of Th1 and Th2 Cell Densities and Th1/Th2 Cytokine Profiles in Colorectal Cancer
Source: Cancer Epidemiol Biomarkers Prev. 2025 Aug 14;34(11):2032–41. doi: 10.1158/1055-9965.EPI-25-0767 (PMC12580825; doi:10.1158/1055-9965.EPI-25-0767)
Supplement: Table S10 — Univariable and multivariable Cox regression models for cancer-specific survival and overall survival according to serum cytokine levels in Cohort 1. [file epi-25-0767_table_s10_suppst10.pdf]

Table S10. Univariable and multivariable Cox regression models for cancer-specific survival and overall survival according to serum cytokine levels in Cohort 1.

		Colorectal cancer-specific survival			Overall survival		
	No. of cases	No. of events	Univariable HR (95% CI)	Multivariable HR (95% CI)	No. of events	Univariable HR (95% CI)	Multivariable HR (95% CI)
**Serum IFNG**							
Low	199	29	1 (referent)	1 (referent)	52	1 (referent)	1 (referent)
Intermediate	200	39	1.40 (0.87-2.27)	1.32 (0.80-2.16)	67	1.38 (0.96-1.98)	1.17 (0.81-1.69)
High	200	31	1.03 (0.62-1.71)	1.11 (0.66-1.87)	62	1.15 (0.79-1.66)	1.10 (0.75-1.60)
P_{Trend}			0.92	0.70		0.50	0.65
**Serum IL12**							
Low	200	31	1 (referent)	1 (referent)	55	1 (referent)	1 (referent)
Intermediate	200	36	1.20 (0.74-1.94)	0.91 (0.55-1.49)	59	1.10 (0.76-1.59)	0.91 (0.63-1.33)
High	199	32	1.08 (0.66-1.77)	0.93 (0.55-1.56)	67	1.28 (0.89-1.82)	1.08 (0.75-1.57)
P_{Trend}			0.75	0.78		0.18	0.64
**Serum IL2**							
Low	200	30	1 (referent)	1 (referent)	49	1 (referent)	1 (referent)
Intermediate	200	32	1.11 (0.67-1.82)	0.94 (0.55-1.62)	63	1.36 (0.93-1.97)	1.42 (0.96-2.10)
High	199	37	1.33 (0.82-2.15)	1.02 (0.62-1.68)	69	1.53 (1.06-2.21)	1.35 (0.93-1.96)
P_{Trend}			0.25	0.91		0.023	0.13
**Serum TNF**							
Low	200	27	1 (referent)	1 (referent)	45	1 (referent)	1 (referent)
Intermediate	200	35	1.37 (0.83-2.27)	1.35 (0.78-2.34)	57	1.35 (0.91-1.99)	1.31 (0.86-1.98)
High	199	37	1.51 (0.92-2.48)	1.21 (0.71-2.07)	79	1.96 (1.36-2.83)	1.49 (1.01-2.20)
P_{Trend}			0.11	0.52		< 0.001	0.045
**Serum IL33**							
Low	200	23	1 (referent)	1 (referent)	51	1 (referent)	1 (referent)
Intermediate	200	44	2.21 (1.29-3.79)	2.09 (1.26-3.47)	68	1.62 (1.11-2.36)	1.50 (1.04-2.15)
High	199	32	1.04 (0.59-1.84)	1.47 (0.86-2.51)	62	1.21 (0.83-1.78)	1.30 (0.90-1.88)
P_{Trend}			0.89	0.19		0.36	0.17
**Serum IL4**							
Low	189	35	1 (referent)	1 (referent)	59	1 (referent)	1 (referent)
Intermediate	190	32	0.92 (0.57-1.49)	0.63 (0.37-1.06)	54	0.95 (0.65-1.37)	0.94 (0.63-1.38)
High	189	30	0.87 (0.54-1.42)	0.61 (0.37-1.01)	62	1.08 (0.75-1.54)	0.87 (0.60-1.25)
P_{Trend}			0.59	0.058		0.68	0.44
**Serum IL5**							
Low	200	40	1 (referent)	1 (referent)	63	1 (referent)	1 (referent)
Intermediate	200	26	0.65 (0.40-1.07)	0.73 (0.44-1.20)	52	0.83 (0.58-1.20)	0.96 (0.66-1.39)
High	199	33	0.84 (0.53-1.33)	0.61 (0.38-0.99)	66	1.06 (0.75-1.50)	0.93 (0.65-1.33)
P_{Trend}			0.42	0.045		0.73	0.70
**Serum IL10**							
Low	200	36	1 (referent)	1 (referent)	58	1 (referent)	1 (referent)
Intermediate	200	28	0.79 (0.48-1.30)	0.71 (0.42-1.20)	57	1.01 (0.70-1.46)	0.97 (0.67-1.41)
High	199	35	1.04 (0.65-1.66)	0.78 (0.48-1.28)	66	1.24 (0.87-1.76)	1.00 (0.69-1.45)
P_{Trend}			0.88	0.32		0.23	0.99
**Serum IL13**							
Low	200	27	1 (referent)	1 (referent)	51	1 (referent)	1 (referent)
Intermediate	200	38	1.50 (0.92-2.46)	1.18 (0.69-2.00)	65	1.39 (0.97-2.01)	1.38 (0.94-2.02)
High	199	34	1.33 (0.80-2.20)	1.04 (0.61-1.78)	65	1.38 (0.95-1.99)	1.32 (0.90-1.93)
P_{Trend}			0.28	0.93		0.092	0.17
**Serum IFNG:IL4 index**							
Low	189	35	1 (referent)	1 (referent)	61	1 (referent)	1 (referent)
Intermediate	190	30	0.84 (0.51-1.36)	0.79 (0.47-1.31)	53	0.86 (0.60-1.25)	0.92 (0.63-1.34)
High	189	32	0.91 (0.56-1.47)	1.35 (0.82-2.21)	61	1.00 (0.70-1.43)	1.21 (0.84-1.74)
P_{Trend}			0.69	0.29		1.00	0.31
**Serum Th1:Th2 produced cytokine index [(IFNG×IL2×TNF):(IL4×IL5×IL10×IL13)]**							
Low	189	33	1 (referent)	1 (referent)	61	1 (referent)	1 (referent)
Intermediate	190	28	0.81 (0.49-1.34)	1.12 (0.66-1.90)	52	0.81 (0.56-1.17)	0.95 (0.65-1.39)
High	189	36	1.04 (0.65-1.67)	1.71 (1.04-2.81)	62	0.96 (0.68-1.37)	1.15 (0.79-1.66)
P_{Trend}			0.86	0.036		0.84	0.47
**Serum Th1:Th2 inducing cytokine index [(IFNG×IL12):(IL4×IL33)]**							
Low	189	34	1 (referent)	1 (referent)	54	1 (referent)	1 (referent)
Intermediate	190	30	0.86 (0.53-1.40)	0.76 (0.46-1.26)	56	1.02 (0.70-1.48)	0.93 (0.64-1.36)
High	189	33	1.00 (0.62-1.62)	1.17 (0.71-1.93)	65	1.26 (0.88-1.81)	1.22 (0.84-1.76)
P_{Trend}			0.99	0.60		0.20	0.28

Abbreviations: CI, confidence interval; HR, hazard ratio

Multivariable Cox proportional hazards regression models were adjusted for sex, age (<65, 65–75, >75), year of operation (2000–2005, 2006–2010, 2011–2015, 2016–2020), tumor location (proximal colon, distal colon, rectum), disease stage (I–II, III, IV), tumor grade (low-grade, high-grade), lymphovascular invasion (negative, positive), mismatch repair (MMR) status (proficient, deficient), *BRAF* status (wild-type, mutant).
